# The genomic footprints of migration: how ancient DNA reveals our history of mobility

**DOI:** 10.1186/s13059-025-03664-w

**Published:** 2025-07-16

**Authors:** Matthew P. Williams, Christian D. Huber

**Affiliations:** Department of Biology, The Pennsylvania State University, University Park, PA 16802 USA

## Abstract

Ancient DNA has emerged as a powerful tool for studying human migration through the detection of admixture signatures. Here, we present the theoretical principles and methodologies for admixture analysis, with an emphasis on *f*-statistics and qpAdm. We review case studies from the literature demonstrating how these methods uncover patterns of human mobility, and discuss challenges related to data quality, demographic complexity, and sample representativeness on admixture and migration inferences. Finally, we highlight promising advancements in admixture analysis and underscore the importance of integrating genetic, archaeological, and historical data to achieve a more interdisciplinary and nuanced reconstruction of human history.

**Supplementary Information:**

The online version contains supplementary material available at 10.1186/s13059-025-03664-w.

## Background

The advent of ancient DNA technology has ushered in a new era in the study of human history, giving rise to the fields of paleogenomics and archaeogenomics — loosely the study of prehistoric and historical genomes, respectively. These subdisciplines have revealed that human populations, including Neanderthals and Denisovans, are not neatly delineated into isolated groups. Rather, we are all related in a complex tapestry of genetic threads, where gene flow is the rule and isolation the exception [1–6]. However, the canonization of ancient DNA’s new human history [7] into the broader field of archaeology has not been without controversy [8–15]. Since the 1960s, archaeology had been moving away from migration-based explanations for changes in material culture [16], adopting the principle that cultural artifacts are not inherently linked to specific populations. This shift in archaeological thinking, encapsulated in the phrase “pots don’t equal people” [17], established an interpretative framework that would become conceptually antagonistic with early ancient DNA analyses published during the mid to late 2010s revealing signatures suggestive of widespread population movements. As such, much of the resulting friction between some geneticists and archaeologists was fueled by divergent perspectives on the significance of migration as explanations for historical events, a tendency of geneticists to treat archaeological and cultural groupings as well-defined categorical entities, as well as mutual misunderstandings and oversimplifications [15, 18–20].

In perhaps what should not have come as a surprise, these early misunderstandings exemplify the difficulties of collaboration between disciplines that possess distinct methodological approaches and epistemological foundations. Although archaeology and paleo/archaeogenomics both study the human past, they operate within separate scientific domains, sometimes employing the same language to describe different — albeit related concepts. For instance, whilst there is no single consensus on a definition of “migration” in archaeology [21], broadly the concept refers to individuals’ movements occurring within or between various geographic locations [22–24]. Conversely, in population genetics, migration refers specifically to the proportion of individuals who have immigrated into a population in the past, with only those migration events that result in integration through mating being informative. It is commonly quantified as the backward migration rate or admixture proportion, representing the probability that a randomly selected individual in the current generation originated from a different population in the previous generation.

The study of human migration necessitates a nuanced understanding of the limitations inherent in both genetic and archaeological methodologies. Genetic data, while informative about biological relationships, lacks intrinsic geographical and cultural context, rendering it indirectly informative about migration patterns. In contrast, archaeological evidence, such as stable isotope signatures, can elucidate recent course geographical origins but are incapable of providing insights into genetic relatedness or inheritance. In addition, whilst historical documents provide invaluable information regarding dynamic cultural and ethnic identity, they are often produced by the elite class — scribes, officials, and religious functionaries — which may limit their broader ability to inform migration histories. As such, each discipline’s unique analytical approaches and data types necessitate a cautious approach to inference-making, ensuring that conclusions derived from genetic data do not inappropriately inform archaeological and historical interpretations, and vice versa. So-called domain-specific inferences [25] are crucial for maintaining the integrity of interdisciplinary research.

In light of these challenges, this paper begins by providing an introduction to the principles underlying genetic admixture and methods commonly employed to identify its signatures in ancient DNA. We then discuss some of the limitations of these methods and their implications for inferring admixture and migration patterns. Subsequently, we review case studies from the ancient DNA literature that highlight different approaches in admixture inference to study historical mobility. Finally, we highlight computational and interpretative advancements poised to enhance our resolution in detecting signatures of admixture and foster a more nuanced, interdisciplinary understanding of past human mobility.

## The admixture process

In population genetics, admixed populations are conceptualized as a linear combination of their distinct sources [26]. This model posits that in the generation following admixture, allele frequencies at any locus in a randomly mating admixed population are weighted averages of the corresponding frequencies in parental populations, with admixture weights determined by their relative parental contributions. Genetic drift in the admixed and source populations following admixture causes random deviations at individual loci, yet on average this relationship persists, highlighting the importance of analyzing numerous independent loci in admixture analysis (readers less familiar with population genetic concepts are directed to Supplementary Note 1, which provides a glossary of key terminology used throughout this manuscript). Under a simplified model of neutrality and admixture confined to a single founding generation, the expected genetic contribution from each source population is solely defined by the initial mixing parameters [27] and remains, on average, unchanged across all subsequent generations as genetic drift is agnostic to the alleles’ ancestral source. Whilst often viewed from the perspective of population-level processes, at the individual level, admixed offspring inherit recombined parental haploid chromosomes that may themselves reflect diverse grandparental origins [28, 29]. The genome-wide admixture fraction thus refers to the proportion of an individual’s genome that traces back to source populations [27, 30].

While these idealized concepts provide a clear theoretical framework for understanding admixture, underlying them is the fundamental question of what constitutes a “population” — is it a real biological entity and if so, how is it defined, bounded, and identified — especially in the context of sparse and often non-contemporaneous sampling characteristic of ancient DNA research. Delineating human groups amid continuous genetic variation increasingly concerns both population genetics [28, 31–34] and humanities research [35–37]. It has been argued that biological populations are best understood as research constructs or as statistical modeling tools that simplify reality [31, 36]. Genetic terminology and population labeling presents related challenges, with recent recommendations advocating phrases like “genetically similar to” over definitive ancestry labels, cautioning against conflating genealogical ancestry, genetic ancestry, and genetic similarity [28, 31] — challenges undoubtedly amplified in ancient DNA contexts [38].

### Principles behind testing for – and quantifying – admixture

Whilst initially inhibited by its inherent limitations of scarcity and degradation [39], ancient DNA is now routinely used to both test for, and estimate admixture proportions in ancient individuals and populations. Here, we present an accessible overview of the fundamental principles and some frequently employed methodologies for detecting admixture in ancient populations with ancient DNA.

### Testing admixture models and estimating proportions with *D*- and *f*-statistics

**Fig. 1:**
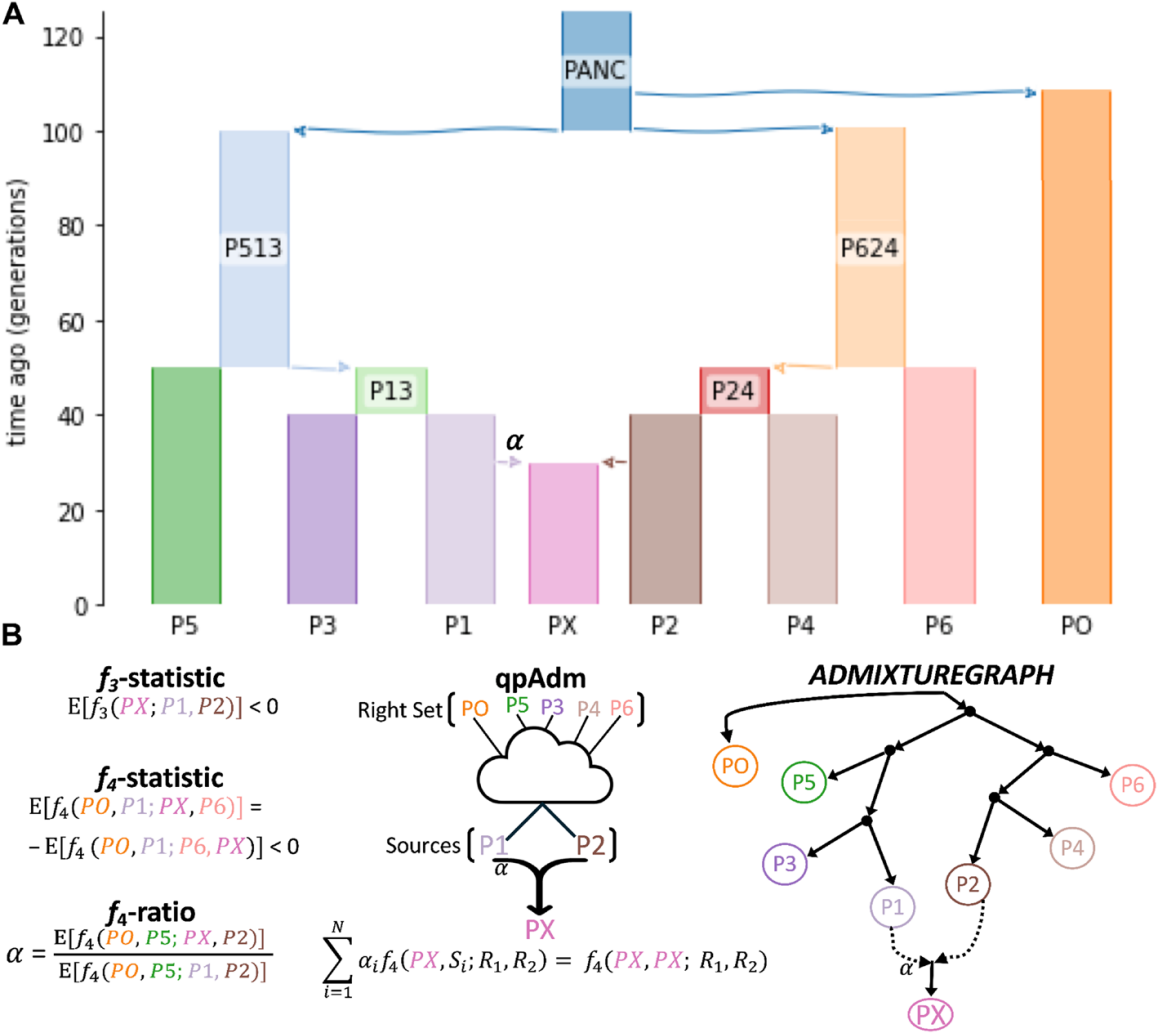
**A** Demographic model used throughout to communicate principles underlying admixture analysis and methods. The population labels in our model are defined as follows: PANC represents the ancestral population from which all sampled populations (PX, PO, and P1-P6) descend; PO functions as an outgroup population, exhibiting approximately equal genetic distance to all other sampled populations; and PX denotes an admixed population whose genetic ancestry derives from source populations P1 and P2 with admixture proportions α and (1 − α), respectively. **B** Illustrative expectations of commonly used admixture inference methods with respect to the above (**A**) demographic model

A suite of methods has gained popularity in the ancient DNA community that both formally test for admixture and estimate the contributing proportions from putative source populations (described below). Whilst these methods differ in their approach, assumptions, and complexity of the demographic history they attempt to infer, most leverage covariances in allele frequency differences between populations estimated from Patterson’s *F*-statistics (hereafter, *f*-statistics) [40–42]. To this end, *f*-statistics have become a foundational tool for researching the admixture history of ancient populations. Within the family of *f*-statistics, the subscript denotes the number of populations included, with *f*_2_ = E[(*p*_1_ – *p*_2_)^2^], *f*_3_ = E[(*p*_X_ − *p*_1_)(*p*_X_ − *p*_2_)], and *f*_4_ = E[(*p*_1_ − *p*_2_)(*p*_3_ − *p*_4_)] analyzing two, three, and four populations, respectively. Below we present an introduction to the theoretical foundations of *f*-statistics, and their practical use in deciphering admixture histories. To guide intuition, throughout we reference a simplified demographic model introduced in Fig. 1A — hereafter Model 1.

#### The ***f***_2_-statistic

**Fig. 2:**
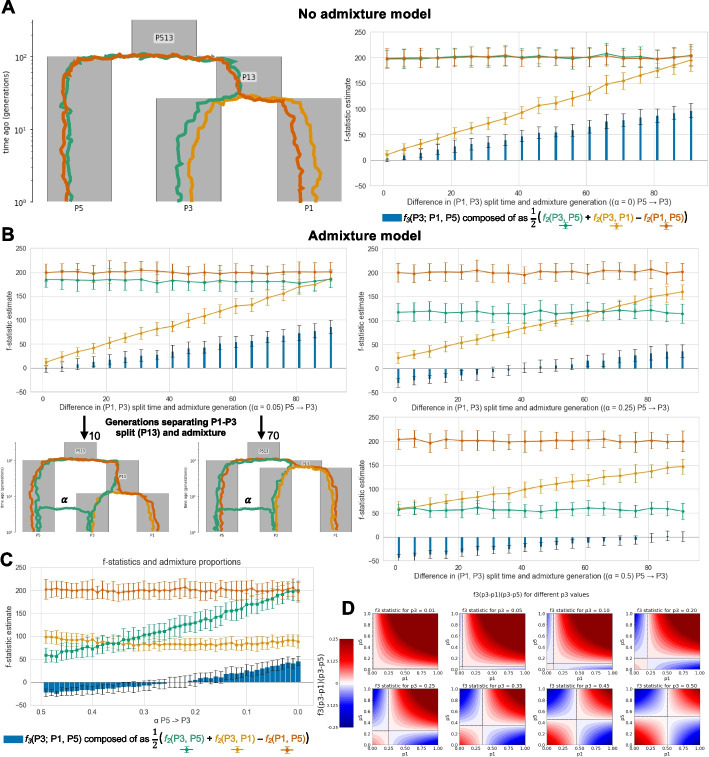
The impact of varying admixture and split-time parameters on the *f*_3_-statistic. **A** A no admixture model with the *y*-axis showing the *f*-statistic estimate for the *f*_3_-statistic (blue bars) computed as the combination of *f*_2_-statistics (green, orange, and yellow line plots). The genetic drift path associated with each *f*_2_-statistic test pair is depicted in the demography figure with the corresponding line color. **B** An admixture model with increasing generations separating the time of admixture from P5 to P3 and the split of populations P3 and P1 (*x*-axis). The three plots correspond to three proportions of admixture (*α* = 0.05, 0.25, and 0.5) from P5 to P3. Plots of the simulated demography (**A **and** B**) were generated by demesdraw 0.4.0 [43]. **C** The same *f*-statistic calculations as **A **and **B** but under a fixed split-time demography (P3, P1 split = 45 gen) with varying proportions of admixture [0, 1] from population P5 to P3 (*x*-axis). All simulations (**A**–**C**) were computed in msprime 1.3.0 [44] with the first 20 generations simulated with the DiscreteTimeWrightFisher model and the remaining generations with the StandardCoalescent using the following genome parameters: genome length (L) = 46,709,983 and recombination rate = (1.72e − 08) taken from human chromosome 21 under the stdpopsim HomSap model ID [45, 46], *N*_e_ = 1000e., sample size = 20 with msprime num_replicates = 50. All *f*-statistic calculations were computed with tskit 0.5.6 mode ='branch'. **D** Expected *f*_3_-statistic values computed as E[(*p*_3_ − *p*_1_)(*p*_3_ − *p*_5_)] where p represents an allele frequency across the range [0, 1] for populations P1 (*x*-axis) and P5 (*y*-axis) for eight different fixed *p*_3_ values (0.01, 0.05, 0.10, 0.20, 0.25, 0.35, 0.45, 0.5) in population P3

The most fundamental of the *f*-statistics, the *f*_2_-statistic, quantifies the amount of genetic drift separating two sampled populations as a measure of the average squared difference in their allele frequencies. As demonstrated in Fig. 2A, the *f*_2_-statistic serves as a quantitative measure of population divergence, with its value increasing in proportion to the amount of genetic drift (a function of generations of separation and effective population size) experienced by the populations. Importantly, independent genetic drift as estimated by the *f*_2_-statistic can be partitioned along branches of a phylogeny (otherwise known as the additivity principle), a feature that distinguishes it from other measures of population differentiation, such as *F*_ST_. As such, for two populations that solely split from a shared ancestor, such as P1 and P3, the genetic drift separating them is equal to the sum of genetic drift along their connected branches: *f*_2_(P1, P3) = *f*_2_(P1, P13) + *f*_2_(P13, P3) — (see Fig. 2A).

Notably, the additivity principle assumes populations are exclusively related through a tree-like population history. Its value lies in its capacity to infer non-tree-like population relationships and, in turn, admixture, by identifying significant deviations between expected and observed *f*_2_-statistics when assuming a tree-like structure. This is because if there are admixture events in the population’s history, genetic relationships cannot be represented by simple tree structures as they harbor divergent histories of genetic drift that trace their own unique paths through the phylogeny. For example, the tree-like evolutionary relationship between the Model 1 outgroup population PO and one of the admixing source populations, P1, exemplifies additivity (see Fig. 1). Here, the genetic drift separating them equals the cumulative drift along their connecting branches, namely *f*_2_(PO, P1) = *f*_2_(P1, P13) + *f*_2_(P13, P513) + *f*_2_(P513, PANC) + *f*_2_(PANC, PO). However, the admixture process systematically reduces the *f*_2_-statistic, resulting in the admixed population PX exhibiting allele frequencies that more closely approximate ancestral frequencies (PO) than either of its source populations (P1 and P2). As a consequence, although PX derives its entire ancestry from P1 and P2 and all have the same population size, its *f*_2_-statistic with PO is reduced below those of both sources (*f*_2_(PO, PX) < *f*_2_(PO, P1) and *f*_2_(PO, P2)), with the maximum reduction occurring at equal source (P1, P2) admixture contributions.

#### The ***f***_3_-statistic

Within the broader family of *f-*statistics, the *f*_3_ can be represented as an algebraic arrangement of the *f*_2_-statistic: *f*_3_(P1; P2, P3) = ½ (*f*_2_(P1, P2) + *f*_2_(P1, P3) − *f*_2_(P2, P3)) [42]. Two common applications of the *f*_3_-statistic in archaeogenetics are to quantify the amount of genetic drift separating populations, and to test if a target population is admixed from two putative sources [41]. Under a no-admixture scenario from Model 1, the *f*_3_-statistic *f*_3_(P3; P1, P5) estimates how much genetic drift has occurred along the terminal branch leading to P3 (Fig. 2A). Viewing the *f*_3_-statistic as a combination of *f*_2_-statistics, i.e., *f*_3_(P3; P1, P5) = ½ (*f*_2_(P3, P1) + *f*_2_(P3, P5) − *f*_2_(P1, P5)), provides a complementary perspective from which to see that the genetic drift separating P1 and P5 is subtracted from their shared drift with P3 to reveal the genetic drift that has occurred exclusively since the split of population P3 from its ancestral population P13 (i.e., equal to *f*_2_(P13, P3)). Importantly, since *f*_3_ corresponds to a terminal branch length under the specific case of a no-admixture scenario, it cannot be negative (i.e., *f*_3_(P3; P1, P5) = *f*_2_(P3, P13) ≥ 0).

To illustrate how the *f*_3_-statistic can identify admixture, imagine individuals from population P5 migrating and intermixing with the individuals from population P3 at some time in the past (Fig. 2B). We first note that genetic drift unique to P5, when introduced to P3 through admixture, decreases the value of the statistic *f*_2_(P3, P5) and breaks the symmetry of P3 and P1 in their relationship with P5 (as *f*_2_(P3, P5) < *f*_2_(P1, P5)) (Fig. 2C). This has the effect of decreasing the value of *f*_3_(P3; P1, P5), such that significantly negative values provide strong evidence that P3 is the product of admixture between sources closely related to P1 and P5. The impact of admixture from P5 to P3 on the *f*_3_-statistic can also be seen by recognizing that admixture from P5 to P3 will drive the expected allele frequency in P3 (*p*_3_) to become intermediate between P5 (*p*_5_) and P1 (*p*_1_) (Fig. 2D). As such, when viewing the *f*_3_-statistic as originally defined in Reich et al. [40], as a covariance of two allele frequency differences *f*_3_(P3; P1, P5) = E[(*p*_3_ − *p*_1_)(*p*_3_ − *p*_5_)], the covariance becomes negative when *p*_3_ values are intermediate between *p*_1_ and *p*_5_ since *f*_3_-statistic negativity implies that a positive (*p*_3_ − *p*_1_) is linked to a negative (*p*_3_ − *p*_5_), or vice versa. Importantly, we note previous work [40–42, 47] has identified specific demographic conditions, such as post-admixture drift, that can result in a positive *f*_3_-statistic even in the presence of admixture (i.e., false negative) which we discuss in subsequent sections below.

#### The ***f***_4_-statistic

The *f*_4_-statistic, initially referred to as the four-population test [40], is another widely used approach for detecting admixture in ancient DNA research. Closely related is the *D*-statistic, originally developed to identify introgression between Neandertals and extant humans [48], with the *f*_4_- and *D*-statistic being the same up to a normalization factor (outlined in Patterson et al. [41]). One of the functions of the *f*_4_- and *D*-statistic in identifying admixture is to conduct a “treeness” test, which determines if four populations have a strictly cladal relationship with at most one internal branch separating them, a condition referred to as the “four-point condition.” The *D*-statistic, colloquially known as “ABBA BABA,” tests the null hypothesis that two sets of populations, such as (P1, P3) and (P2, P4), form clades relative to each other by quantifying the difference between two allele sharing patterns: (ABBA), which pairs populations two and three together based on their shared allele, and (BABA), where the inverse pattern is observed, standardized by the sum of those patterns (when applied to single nucleotide polymorphism (SNP) data, as is typically used in ancient DNA, these patterns are computed on allele frequencies [49]). Under the null hypothesis of no admixture, the two patterns are solely found due to incomplete lineage sorting in the ancestral population of P1, P2, and P3 (i.e., PANC from Model 1). Since coalescence of the P1, P2, and P3 lineages in this ancestral population are random, the frequency of the two ABBA/BABA patterns at bi-allelic sites are expected to be equal, leading to a *D*/*f*_4_-statistic of zero.

To gain intuition into how the *D*/*f*_4_-statistic tests for the presence of admixture, we now imagine an admixed population, PX, formed as a mixture of P1 and P2 sources, as shown in Model 1. The configuration of the *f*_4_-statistic “treeness” test as *f*_4_(P1, PX; P2, P4) will test the null hypothesis that (P1, PX) and (P2, P4) form distinct phylogenetic clades with the null hypothesis expectation *f*_4_(P1, PX; P2, P4) = E[(*p*_1_ − *p*_X_) (*p*_2_ − *p*_4_)] = 0. However, admixture from P2 to PX pulls PX’s allele frequency closer to P2’s, such that the allele frequency difference between P1 and PX is no longer independent of the allele frequency difference between P2 and P4. As a result, the covariance in allele frequency differences between (P1, PX) and (P2, P4) will become negative, and thus the null hypothesis of cladality will be rejected such that *f*_4_(P1, PX; P2, P4) < 0. We also note that the *f*_2_ formulation of the *f*_4_-statistic in our above example, *f*_4_(P1, PX; P2, P4) = ½ (*f*_2_(P1, P4) + *f*_2_(PX, P2) − *f*_2_(P1, P2) − *f*_2_(PX, P4)), reveals that admixture from P2 to PX reduces their allele frequency differentiation such that *f*_2_(PX, P2) < *f*_2_(PX, P4) which in turn, under favorable demographic conditions discussed further below, results in a negative *f*_4_-statistic and an overrepresentation of ABBA patterns relative to BABA patterns in the *D*-statistic formulation (see Supplementary Note 2 for the algebraic derivation of the *f*_4_-statistic as composed of as *f*_2_-statistics).

Importantly, in the absence of admixture at least one of the D/*f*_4_-statistic values will be zero (i.e., *f*_4_(P1, P3; P2, P4) = 0), and the others will have absolute values different from zero (i.e., *f*_4_(P3, P2; P1, P4) > 0 = |*f*_4_(P2, P3; P1, P4)| = |*f*_4_(P3, P2; P4, P1)|) [50]. In practice, an outgroup population (e.g., PO in Model 1) is typically included to polarize the test statistic and help identify the populations contributing to its significance [41]. Complex evolutionary history, however, complicates simplistic interpretations of admixture history based solely on *D*- and *f*_4_-statistics, as these values function as point estimates representing the average across diverse phylogenetic pathways within a population’s demographic history [51].

### Estimating admixture proportions with the *f*_4_-ratio statistic

After a population of interest is confirmed to be admixed from putative sources, the next phase of analysis involves estimating its admixture proportions with respect to a set of candidate source populations. The *f*_4_-ratio statistic, first described as the *f*_4_-ancestry estimation by Reich et al. [40], offers a robust approach for determining the ancestral proportions present in a target population or individual that can be traced back to the source population (Fig. 1B). A comparable methodology was utilized by Green et al. [48] to estimate Neanderthal genetic contributions in extant non-African populations.

To elucidate the relationship between ratios of *f*_4_-statistics (i.e., the *f*_4_-ratio) and admixture proportions, we recall that in randomly mating admixed populations, expected allele frequencies are linear combinations of source population frequencies under the assumption of neutrality, and that we can ignore post-admixture drift. The coefficients in these combinations represent the admixture fractions, thus establishing a conceptual link between observed allele frequencies and the underlying admixture process. We now return to Model 1 (Fig. 1A) in which the allele frequency (*p*_X_) in the admixed population (PX) is expressed as a linear combination of the source population (P1 and P2) frequencies (*p*_1_ and *p*_2_); ignoring post-admixture genetic drift and assuming neutrality. The admixture proportion (α) serves as the weighting factor, such that: *p*_X_ = α*p*_1_ + (1 − α)*p*_2_. From this, the *f*_4_-statistic involving population PX is derived exactly such that:

*f*_4_(PO, P5; PX, P4) = *f*_4_(*p*_O_, *p*_5_; *p*_X_ *p*_4_) = *f*_4_(*p*_O_, *p*_5_; (α*p*_1_ + (1 − α)*p*_2_), *p*_4_) = α*f*_4_(*p*_O_, *p*_5_; *p*_*1*_, *p*_4_) + (1 − α)*f*_4_(*p*_O_, *p*_5_; *p*_2_, *p*_4_), revealing the *f*_4_-statistic of an admixed population is equivalent to the *f*_4_-statistics of its sources, weighted by their admixture coefficient [52]. Consequently, it also follows that *f*_4_(PO, P5; PX, P2) is equivalent to α*f*_4_(PO, P5; P1, P2), since *f*_4_(PO, P5; P2, P2) is by definition zero. From here, we can use the ratio of two *f*_4_-statistics to algebraically derive the admixture proportion (α) of P1 to PX, in the form α = *f*_4_(PO, P5; PX, P2)/*f*_4_(PO, P5; P1, P2), revealing the link between allele frequencies, *f*_4_-statistics, and admixture proportions (Fig. 1B).

### Testing admixture models and estimating proportions with qpAdm

First introduced in Haak et al. [52], qpAdm leverages the principles behind both the *f*_4_-ratio and the *f*_4_-statistic to statistically test proposed models of admixture and provide estimated contributions from proposed sources to a target population of interest (Fig. 1B). Whilst the complex phylogenetic relationships between the target, source, and reference populations are not explicitly modeled in qpAdm, this in turn provides significant flexibility in practice. By using *f*-statistics to capture patterns of shared genetic drift between the target, souce, and reference populations, qpAdm is able to formally test the hypothesis that the putative source populations are the sole contributors to the target population’s ancestry (i.e., none of the reference populations contribute additional gene flow to the target that is not captured in the sources) without an explicit knowledge of the phylogenetic relationship between the source and reference populations. Although a detailed explanation of qpAdm is beyond the scope of this paper (see SI. 9 and 10 of Haak et al. [52], in addition to SI qpAdm User Guide and Supplementary Materials 2 of Harney et al. [53]), we provide a brief summary of how qpAdm employs principles from both the *f*_4_-statistic and *f*_4_-ratio to perform admixture analyses (also see Supplementary Note 3 for an introductory overview on the qpAdm theory and implementation).

When running qpAdm, a user will input three key population variables: a target population of interest, a set of putative source populations, and a set of reference populations selected to capture the genetic diversity of the region and time period. From the perspective of their positions in the *f*_4_-statistic, the target and source populations are commonly referred to as the “left-set” and the reference populations the “right-set”. With the left and right-group population sets, qpAdm generates matrices of *f*_4_-statistics which serve as the foundation for computing the model *p*-value and estimating admixture proportions from each of the candidate sources. Recalling from the above section: *The f*_*4*_*-statistic*, if two sets of populations form clades, e.g., (P1, P3) and (P2, P4), then *f*_4_(P1, P3; P2, P4) has expectation zero, indicating only a single branch connects the clades. In contrast, under an admixture model, *f*_4_(P1, PX; P2, P4) < 0 indicating there is at least more than one branch connecting the two population sets (P1, PX) and (P2, P4) due PX sharing unique genetic drift with P2.

In fact, the number of branches connecting the left and right population sets underlies the principle of the *f*_4_-statistic matrix rank first introduced in Reich et al. [54] and Moorjani et al. [55] and is the basis of the qpWave software. In practice, the *f*_4_-statistic matrices used by qpAdm is formed by fixing one population in the left population list (in practice the target population) and one population from the reference population set (in practice the population with the best genetic coverage), forming a matrix of dimensions (*n*_L_ − 1)(*n*_R_ − 1). It was shown in Reich et al. [54] and Moorjani et al. [55] that when fixing one population from the left set and one from the right set, the maximum rank of the matrix is *n*_L_ − 1. The rank in this instance refers to the minimum number of branches connecting the two sets of populations. When the rank of the *f*_4_-statistic matrix encompassing both sources and target in the left set does not exceed that of the source-only matrix, it indicates that the sources’ genetic diversity fully captures the target’s ancestry, within the resolution of the right group.

To account for linkage disequilibrium — i.e., the correlation in genealogical history for SNPs located in close physical proximity along a chromosome — qpAdm implements a block jackknife resampling approach, dividing the genome into blocks and sequentially recalculating *f*_4_-statistics while omitting one block at a time. This method both accounts for the non-independence of demographic histories along the genome and quantifies the errors and uncertainty associated with each individual *f*_4_-statistic. In addition, qpAdm implements a likelihood ratio test to statistically test whether the target population’s ancestry is solely explained by the proposed admixture sources or requires a more complex model incorporating additional independent admixture from a population in the right-group. A significant *p*-value (typically *p* < 0.05 or 0.01) indicates that the null-hypothesis (i.e., the simpler model of the target solely explained by the proposed admixture sources) is rejected in favor of the more complex model. Importantly then, a large *p*-value indicates not that a plausible qpAdm model is statistically significant, but rather that it cannot be rejected based on the current data. In addition to a *p*-value, qpAdm will also generate an admixture proportion, which is typically only interpreted for plausible models, with weights outside of the biologically relevant ranges [0, 1] being one criteria used to reject proposed admixture models.

To estimate the admixture proportion, qpAdm extends the principles of the *f*_4_-ratio outlined above, whereby if an admixed target population of interest has ancestry related to *N* different source populations, then the *f*_4_-statistic of the admixed target population can be composed as a linear weight of the *f*_4_-statistic of its sources:

${\sum }_{i=1}^{N}{\alpha }_{i}{f}_{4}(T, {S}_{i}; {R}_{1}, {R}_{2}) = f4(T, T; {R}_{1}, {R}_{2}) = 0$ (equation modified from page 129 of Haak et al. [52] where *T* represents the target population, *R*_1,2_ qpAdm right populations, and *S*_*i*_ qpAdm source populations). Thus, the qpAdm admixture proportion estimates emerge from the requirement that the *f*_4_-statistics involving the source populations need to be weighted and added up in a way such that they equal the *f*_4_-statistics involving the target.

### Modeling complex admixture histories with graphs

While qpAdm’s minimal model phylogeny allows for more flexibility, the absence of explicitly modeled relationships among all populations can limit the interpretability of the findings. In contrast, the ADMIXTUREGRAPH (AG) (Fig. 1B) framework [40] attempts to provide a comprehensive model of the phylogenetic and admixture relationships within a set of populations. An AG structure consists of an ordering of population splits, positions of admixture events, branch length parameters, and mixture proportions. However, the AG framework remains a reduced representation of a complete demographic model [56], as it does not include estimates of population sizes, nor population split or admixture times. Given the branch lengths and ordering of population splits and admixture events defined in the AG, admixture proportions (α) form part of a more explicit model of the populations’ demographic history. Various software packages are available for admixture graph (AG) analysis, where they differ in their level of automation and approaches to defining the initial AG configuration, exploration of alternative models, and assessment of the model fit to empirical allele frequency data. However, the *f*-statistics framework remains central to all of AG tools (with the exception of *TreeMix*).

## Challenges in admixture analysis and interpretation using ancient DNA

Whilst ancient DNA has revolutionized our understanding of the human past, reconstructing the admixture history of ancient populations is not without its challenges. Both low-quality genetic data and aspects of demography can limit the statistical power of population genetic inferences from ancient DNA. Moreover, interpreting admixture signatures within the context of historical processes and mechanisms of migration and mating presents a significant challenge to unifying archaeological, historical, and genetic analyses. In the following discussion, we provide a concise overview of some of the key challenges in ancient DNA research for reconstructing admixture histories and inferring their underlying mechanisms, and discuss current approaches in the field for identifying signatures of human mobility from admixture signatures.

### DNA degradation

One of the most fundamental limitations in ancient DNA research is overcoming the impacts of degradation due to post-mortem DNA damage on the quantity and quality of DNA [57]. Both time and the environment contribute to the breakdown of DNA molecules, resulting in highly fragmented and short DNA reads (~ 10–150 bp) [58], in addition to characteristic post-mortem deamination (C → T misincorporations are elevated at the 5′ end of DNA fragments [59]) to the base-pair sequence. The relationship between allele frequency and admixture proportion described above underscores the importance of obtaining unbiased allele frequency estimates for accurate admixture inference. This presents significant challenges throughout the admixture inference process, as the distinctive characteristics of ancient DNA can potentially introduce bias at various stages of analysis. For instance, the impact of ancient DNA damage on admixture inference through qpAdm has been demonstrated by Harney et al. [53] revealing that biases in the estimated admixture proportions can occur with the co-analysis of data with (ancient DNA) and without (present-day) damage patterns, recommending against this strategy.

The scarcity of endogenous DNA in ancient remains has necessitated the development of targeted “capture” technologies for pre-selected (ascertained) SNPs to increase the proportion of sequenced endogenous DNA [41, 52, 60, 61]. Due to the bias towards higher-frequency alleles introduced by the ascertainment process, “captured” ancient DNA datasets do not allow for the utilization of powerful demographic methods that depend on an unbiased site frequency spectrum (SFS) produced by whole-genome sequencing data. As a result, ancient DNA admixture analyses are typically reliant on methods that evaluate the less informative covariance in allele frequency differences (*D*/*f*-statistics) described above. In addition, “low-coverage” specimens, i.e., those which retain only a small fraction of sequenced ascertained SNPs, result in increased standard errors and decreased power to reject false and suboptimal admixture models in qpAdm analyses [47, 53]. Importantly, however, while lower coverage reduces qpAdm power, it does not appear to inherently bias admixture estimates [53]. An additional challenge in working with captured data is that hybridization technologies can introduce potential biases when co-analyzing genomes generated from different platforms and capture-free “shotgun” DNA sequencing approaches [62, 63]. Whilst various strategies permitting the co-analysis of ancient DNA across capture technologies have been developed [62, 63], and some technologies show no observable technical bias [63, 64], researchers should exercise caution when integrating ancient DNA data from different technological sources as SNP capture has demonstrated biases in co-analyses across human populations, particularly when modeling multiple sub-Saharan African populations or archaic human groups like Neanderthals and Denisovans [65].

Due to the fragmentation process, short DNA molecules pose significant challenges in and of themselves for accurate mapping to reference genomes [66]. One of the primary challenges in mapping short DNA molecules is overcoming reference bias, where DNA fragments carrying the reference allele are more likely to map successfully to the human reference genome, with the magnitude of the bias shown to be inversely proportional to read length [67]. Because of the heterogeneous composition of the human reference genome, this could result in both genome-wide and local ancestry bias [68], with varying degrees of impact on different admixture inference and genotype calling methodologies [69]. Importantly, amongst commonly used ancient DNA admixture analysis pipelines, pseudohaploid genotype calling and qpAdm appear to be one of the most robust to reference bias [69]. Future advancements, such as including the adoption of variation graphs over linear references in mapping [68], the use of algorithms to mask out sites vulnerable to ancient DNA characteristic damage from genotyping [70], or correcting genotype likelihoods based on empirical data [69] are promising approaches for mitigating reference bias.

### Demographic history

Demographic histories that result in minimally differentiated populations present additional challenges to admixture reconstruction, compounding the DNA preservation limitations outlined above and effectively reducing admixture detection power — a topic we explore below.

#### Demography and ***f***-statistic power 

**Fig. 3:**
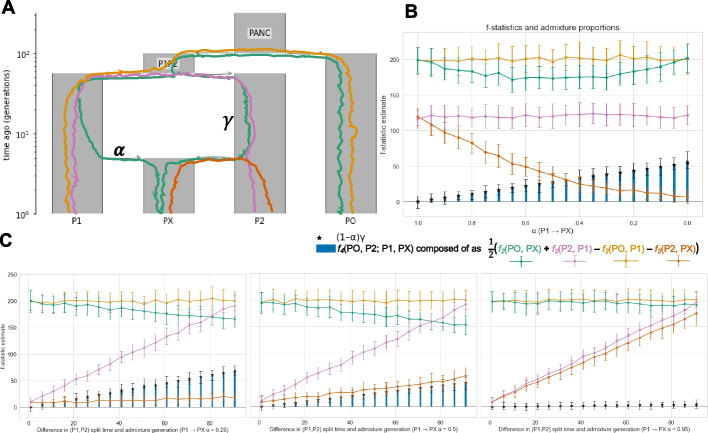
The impact of varying admixture and split-time parameters on the *f*_4_-statistic. All simulations (**B**–**C**) were computed in msprime 1.3.0 [44] with the first 20 generations simulated with the DiscreteTimeWrightFisher model and the remaining generations with the StandardCoalescent using the following genome parameters: genome length (L) = 46,709,983 and recombination rate = (1.72e − 08) taken from human chromosome 21 under the stdpopsim HomSap model ID [45, 46], Ne = 1,000e^2^, and sample size = 20 with msprime num_replicates = 50. **B** The value for the *f*_4_-statistic (blue bars) computed as the combination of *f*_2_-statistics (yellow, pink, green and orange line plots) under a fixed split-time demography (P1P2, PO split = 90 gens) with varying proportions of admixture [0, 1] from population P1 to PX (*x*-axis). **C** The same *f*-statistic calculations as **B** but for three fixed admixture parameters (0.25, 0.5, and 0.95) with the *y*-axis showing the *f*-statistic value for the *f*_4_-statistic (blue bars) computed as the combination of *f*_2_-statistics with increasing generations separating the time of PX formation and the split of populations P2 and P1 (*x*-axis). The genetic drift path associated with each *f*_2_-statistic test pair is depicted in the demography figure (**A**) with the corresponding color. Plots of the simulated demography (**A**) were generated by demesdraw 0.4.0 [43]

As described above, when populations diverge from a common ancestor, they undergo independent genetic drift, causing their allele frequencies to diverge over time. The rate of differentiation is shaped by biological and demographic factors, including the timing of population splits, effective population sizes, and mutation rates. Given the use of allele frequencies in calculating *f*-statistics, their power for inferring demographic history and admixture events is thus directly modulated by these factors. For instance, the *f*_3_-statistic has its greatest power for detecting admixture under the conditions of near-equal contributions from source populations, a large number of generations separating the sources from each other at the admixture event, and limited genetic drift in the admixed population [40, 42]. Importantly for the study of complex demographic history from ancient DNA, Williams et al. [47] demonstrate that both admixture between the sources following their split, and the use of diverged proxy sources — scenarios undoubtedly encountered in empirical human archaeogenetic studies — constrain the range of demographic parameters that can result in a negative *f*_3_-statistic, possibly leading to false negative results in ancient DNA analyses. Similarly, the use of the *f*_4_-statistic to detect admixture also faces statistical limitations under certain demographic scenarios. In Model 1, the covariance in the allele frequency differences between (PX and P2) from (P1 and PO) is determined by the admixture proportion (1 − α) and the evolutionary distance separating P2 from the shared ancestor of P1 and P2 (γ) [50] (Fig. 3A). As the value of this compound parameter (1 − α)γ decreases, the statistic *f*_4_(PO, P2; P1, PX) approaches zero, reducing the signal to detect admixture from P2 to PX (Fig. 3B). Under conditions where γ is small — indicating minimal independent drift between source populations (P1, P2) — the resulting decrease in the compound parameter reduces the value of the *f*_4_-statistic, such that estimates not significantly different from zero suggest P1 and PX form a clade relative to P2, even in the presence of substantial admixture from P2 to PX (Fig. 3C).

Because of its reliance on *f*-statistics, in theory qpAdm is similarly impacted by the demographic and biological constraints described above. Extensive simulations of qpAdm on admixture-graph-like models (similar to that displayed in Fig. 1A) confirm that population divergence is indeed the primary factor limiting its statistical power [47]. Rotating qpAdm approaches [53] (where populations are utilized as both sources and right-groups) for studying admixture in ancient DNA are most effective in identifying a minimal number of plausible models closely related to the true model when population divergence is greater than that of Eurasian Bronze Age (i.e., median pairwise *F*_ST_ > ~ 0.008) with a marked decline in performance towards the lower ends of human population differentiation (i.e., median pairwise *F*_ST_ < ~ 0.003) [47]. Despite challenges in studying historical admixture dynamics among minimally differentiated populations, broad inferences about potential admixing sources remain feasible, as sources, or ancestries, overrepresented in plausible models tend to be more closely related to the true admixing source, regardless of data quality [47, 53]. For high-coverage ancient DNA data, qpAdm *p*-value rankings may provide a means of differentiating between multiple plausible models, with simulations demonstrating that when comparing plausible models larger *p*-values are indicative that a model is closer to the simulated truth [47] — a feature utilized in recent research [71, 72] through the use of qpWave *p*-values as a distance measure to perform individual-based clustering.

Importantly, so long as qpAdm model assumptions are met (we discuss the impact of model violations below), the approach is remarkably effective in accurately detecting and quantifying admixture, with its primary limitations being proportional to the precision of *f*-statistic estimation. In simulations, the use of whole-genome branch-length *f*_2_-statistics to compute qpAdm’s *f*_4_-statistic matrix, i.e., a hypothetical scenario where *f*_2_-statistics could be calculated without estimation error, has shown to significantly enhance its power [47]. This finding suggests that more accurate *f*_2_-statistic estimates from empirical data could further increase qpAdm’s power in resolving historical admixture questions, even among very closely related populations. This raises the prospects for future empirical advancements — e.g., making more efficient use of the data using tree sequence methods — a concept we will revisit in a subsequent section.

#### Continuous migration landscapes

Thus far, our discussion of admixture histories has been restricted to single-pulse events between independently evolving populations. However, much of our recent human demographic history appears to look more like a complex network of ancient communities connected through an evolving web of migration and isolation [7, 72–74]. Indeed, ancient DNA research has illuminated aspects of this complexity, particularly in Eurasia, revealing that the observed correlation between increasing distance from east Africa and decrease in present-day genetic diversity is not simply due to a serial founder effect following an out-of-Africa migration, but rather results from numerous localized admixture events in the Holocene [5].

In and of themselves, *f*-statistics do not make assumptions regarding the migration mechanisms responsible for correlations in allele frequency differences between populations. To this end, Peter [42] demonstrated that the expected values of *f*-statistics differ depending on the underlying model of population structure (including island, serial founder, and stepping stone models, as well as their respective hierarchical variants). For instance, a negative *f*_4_(P1, P2; P3, P4) prima facie suggests the rejection of the null-model of cladality between (P1, P2) and (P3, P4) due to unique shared genetic drift between populations (P2 and P3) and/or (P1 and P4). Indeed, under a one-dimensional stepping-stone model [75] where populations P1, P2, P3, and P4 are arranged linearly with one-way migration rates (*M* →), the expectation of *f*_4_(P1, P2; P3, P4) = $-\frac{8}{7M}$ [42] is negative, asymptotically approaching zero as migration approaches one (Fig. S1). However, if populations P1-P4 are in-fact each composed of sub-demes with one-way migration between them, described as a hierarchical stepping-stone model, the expectation of *f*_4_(P1, P2; P3, P4) = $\frac{14}{55M}$ [42] becomes positive (decreasing exponentially as *M* approaches one), with the prima facie interpretation of a positive *f*_4_(P1, P2; P3, P4) being unique shared genetic drift between (P2 and P4) and/or (P1 and P3) causing the rejection of the null-model of (P1, P2) (P3, P4) as clades (Fig. S1). Moreover, under the hierarchical stepping-stone model the value of the statistic *f*_3_(P2; P1, P3) = $-\frac{0.06}{M}$ [42], begins negative and asymptotically increases towards zero as M approaches one, counter intuitively suggesting that under low levels of migration population P2 can be modeled as originating from an admixture of populations P1 and P3 (Fig. S1). Together, these examples demonstrate the challenges of interpreting demographic and admixture histories from *f*-statistics alone under stepping-stone landscapes.

Given its use of *f*_4_-statistics, qpAdm can also be challenged in accurately modeling the admixture history of populations connected by stepping-stone landscapes. In particular, these models make it difficult to avoid violating the qpAdm assumption of no right-to-left or left-to-right gene flow events following the formation of the target lineage [53, 76, 77]. Using Model 1, we can illustrate the impact of admixture from a right-group to the target population between its formation and sampling on the *f*_4_-statistic — and by extension, qpAdm. One of the statistics in the qpAdm *f*_4_-statistic matrix modeling the genetic history of PX as a mixture of P1 and P2 will be *f*_4_(PX, P2; PO, P6). Under Model 1, the shared drift between P2 and P6 will result in a positive value. However, admixture from P6 to PX following its formation and prior to sampling can result in *f*_4_(PX, P2; PO, P6) being negative. Importantly, admixture from PX to P6 prior to the sampling of P6 will result in the same negative value of *f*_4_(PX, P2; PO, P6), highlighting the absence of admixture directionality information in a single *f*_4_-statistic.

Whilst the precise range of demographic parameters that will result in statistically significant model violations has yet to be explored (see Flegontova et al. [76] for the most comprehensive study using simulations of both two-dimensional stepping-stone landscapes and admixture-graph like models), violations such as these can result in an increase of the *f*_4_-statistic matrix rank (from rank = 1 under no model violations to rank = 2 with admixture from P6 to PX and vise-versa—see above section “*Testing admixture models and estimating proportions with qpAdm*” and Supplementary Note 3), and thus rejection of the “simpler” qpAdm (P1, P2) model. However, the implications of qpAdm model violations illustrated above on admixture inference are nuanced. In expectation, admixture from P6 to PX prior to its sampling will yield a statistically plausible qpAdm 3-source model (P1, P2, P6). However, depending on what aspect of the genetic history of PX one is interested in understanding. i.e., the period corresponding to its formation or their ancestry (as a mixture of P1 and P2), or its cumulative ancestry at the time of sampling (as a mixture of P1, P2, and P6) will influence how accurately a researcher considers the resulting model depicts the genetic history of PX. Similarly, when admixture occurs from PX to P6, the same three-source model is expected to be classified as statistically plausible (i.e., the expectation of a non-significant *p*-value for an increase in qpAdm matrix rank), yet this results in an incorrect interpretation of PX’s genetic history if qpAdm yields a positive non-zero admixture weight from P6 to PX. As such we propose classifying model violations into two camps; those resulting in the plausible inclusion of populations that in reality did not contribute ancestry as “false-inference” model violations, while those that would result in the inclusion of sources not involved in the admixture period of interest — for instance a plausible three-source model when only interested in the origin of PX — “misleading-inference” model violations.

Evaluations of the performance of qpAdm under simulated one-dimensional stepping-stone models [75] were conducted by Harney et al. [53] who modeled populations (named P1–P5) under a continuous symmetric migration model and showed that under a very high migration rate (*M* = 0.01; equivalent to 1% in each generation in a chosen deme coming from each neighboring deme) the “middle” population, i.e., P2, will frequently be plausibly modeled as a 50% mixture of its adjacent populations (P1 and P3). However, under lower migration rates (*M* = 0.001 and 0.0001), the qpAdm model of P2 with P1 and P3 as sources is consistently rejected, whilst the admixture estimate remained roughly 50%. Encouragingly, when simulating effectively the same one-dimensional stepping-stone demographic model as Harney et al. [53], Speidel et al. [77], using *f*-statistics estimated from genealogies with a generational time cut-off (discussed further below), demonstrate an increased power to accurately model admixture under continuous migration at rates *M* = 0.001 and 0.005.

Flegontova et al. [76] extended the evaluation of qpAdm using a demographic model of multiple panmictic demes evolving on an roughly circular two-dimensional landscape over ~ 2500 generations with non-uniform bidirectional gene flows. Their study reveals a disconcerting mismatch between qpAdm *p*-values and the optimal model consisting of the closest sources to the target population. Consequently, on their simulated complex stepping-stone landscape that was sampled either randomly or systematically, right-to-left and left-to-right gene flow events resulted in qpAdm’s tendency to favor geographically distant sources and reject nearby ones leading to a false impression of long-distance migration. Their study also raised concerns regarding low pre-study odds when exploring all possible source combinations on a stepping-stone landscape, wherein the proportion of models tested is dominated by non-optimal models — including sources distant from the target and positioned at small angular distances from each other — effectively creating a systematic bias against qpAdm success. Although qpAdm rejects most non-optimal models, a fraction inevitably remains unrejected, driving false discovery rates above 50% across substantial portions of the parameter space. Consequently, qpAdm protocols that evaluate hundreds to thousands of models per-target while aiming to identify only a few “fitting” ones may produce unreliable results.

### Case studies in ancient DNA admixture analysis and migration inference

Notwithstanding the challenges outlined above, the utility of ancient DNA in studying human mobility hinges on the basic assumption that migration leads to detectable changes in population genetic ancestry. As such, when discussing examples of migration inferred from admixture signatures, it is important to recognize that this approach can miss “hidden” migrations that fail to alter ancestry detectably, either due to genetic similarities between migrant and recipient populations or because migrants did not reproduce in their new location (or remain unsampled). In the following sections, we review a selection of ancient DNA literature that demonstrates a range of approaches of using admixture to detect population migrations and shifts in mobility.

#### Detecting mobility from signatures of genetic discontinuity

Among the earliest and most comprehensively studied uses of ancient DNA for identifying human migration is the expansion of Neolithic farmers from the Near East to Europe. Whilst the Neolithic agricultural expansion from Southwest Asia to Europe was known to archaeologists as early as 1925 [78], its genetic impact on the local European populations remained unclear until a suite of whole-genome ancient DNA studies published between 2012 and 2018 [52, 60, 74, 79–88] unveiled a complex interplay of expansion, admixture, and coexistence between Near Eastern farmers and European hunter-gatherers. Early evidence of complex migration interactions between European hunter-gatherers and farmers came from Skoglund et al. [80] where they analyzed ancient DNA from three key samples: a post-agricultural Scandinavian hunter-gatherer (Ajvide58), a pre-agricultural Iberian hunter-gatherer (LaBrana1), and an early Scandinavian farmer (Gökhem2). Through *D*-statistics, they showed that while the ancestors of Neolithic European farmers had admixed with hunter-gatherers (D(Yoruba, Ajvide58; Gökhem2; Sardinian) *Z*-score < − 6.66), European hunter-gatherer ancestors did not admix with European farmers (|D(Yoruba, Gökhem2; Ajvide58; LaBrana1) *Z*-score|< 1.5), despite at least 40 generations of cohabitation in Scandinavia. In addition, early evidence of distinct migration paths into Europe from the Near East was revealed by Hofmanová et al. [86] using ancient DNA from Aegean Neolithic farmers and various European farming communities. Among other signatures, the *f*_4_-statistic (*f*_4_(Central European-farmers, Spanish Farmers; Aegean Neolithic, ‡Khomani San) *Z*-score < − 3) — revealing that Aegean Neolithic individuals shared more genetic drift with Spanish farmers than with Central European farmers — supported the hypothesis of independent migration routes from the Aegean to Southwestern and Central Europe. Notably, the study’s authors contend that their data delivered the “coup de grâce” to the hypothesis that agriculture spread into and across Europe solely, or primarily, through ideological diffusion and without significant human movement — a perspective we will reevaluate in a subsequent discussion below.

#### Detecting mobility from genetic outliers

While the 25–30,000-year divergence between European hunter-gatherers and Near Eastern farmers [85, 89] yielded genetically distinct populations distinguishable through *D*/*f*-statistics, one may question the utility of ancient DNA in detecting migration between populations of relatively stable structure and less diverged — as observed in Europe and the Mediterranean over the last three millennia [52, 82]. In their analysis of British Bronze and Iron Age genetic history, Patterson et al. [90] developed an approach to detecting periods of increased migration rates based, in-part, on the identification of an elevated proportion of genetic outliers. Using qpAdm to quantify the proportion of Early European Neolithic farmer (EEF), Western European Mesolithic hunter-gatherer (WHG), and Yamnaya Steppe pastoralist (Steppe) ancestry in English and Welsh individuals dating between 2450 BC to 43 AD, Patterson et al. [90] identified 17% of individuals to be EEF-ancestry outliers in both the Chalcolithic/Early Bronze Age (C/EBA — 2450 and 1800 BC), and Middle to late Bronze Age (M-LBA — 1300 and 750 BC) periods.

The use of genetic outliers as a signature of periods of high mobility among genetically similar populations was also employed by Antonio et al. [72] in their study of historical mobility and population structure across Europe, the Mediterranean, and sub-regions of Southwest Asia. In studying three sub-periods from 10 000 BCE to 1950 CE, the authors revealed a striking degree of ancestry heterogeneity, particularly in the regions of Sardinia, the Levant and Egypt, Eastern Europe and the Steppe, and Italy. Employing a qpAdm approach — slightly different to that of Patterson et al. [90]— they estimate 11% of individuals as outliers and 7% putative first-generation migrants, culminating in a detailed admixture network map connecting Europe, the Mediterranean, North Africa, the Levant, and Caucasus.

#### Detecting mobility from measures of population genetic diversity and divergence

In addition to examining ancestry shifts and genetic outliers, summary statistics measuring genetic diversity (such as *F*_ST_) and genetic distance (such as 1 − outgroup *f*_3_-statistic) have also been utilized to identify periods of mobility and admixture. In their study of historical mobility across Europe, the Mediterranean, and sub-regions of Southwest Asia described above, Antonio et al. [72] document a striking observation: despite their detection of high mobility, when computing *F*_ST_ across groups on a sliding spatial grid for each historical period and relating it to mean geographic distance, it remained stable from around the Bronze Age (~ 2300 BCE) onwards, suggesting relatively unchanged genetic differentiation across space and time down to the present day. Through simulations of Wright-Fisher populations evolving neutrally in continuous space, the authors confirmed that even with long-range dispersal as low as 4%, *F*_ST_ decreases over 120 generations, a result inconsistent with their qpAdm outlier-based estimation of ~ 7–11% long-range historical migration. To explain these seemingly incompatible findings, the authors proposed a “transient mobility” hypothesis, which suggests that whilst technological and political developments during the historical period facilitated increased migration, this was not followed by a sustained integration into the local population, resulting in a separation of movement and reproduction compared to prehistoric eras. While emphasizing the need for additional ancient DNA data from urban and rural contexts throughout the historical period to fully assess their hypothesis, the authors also highlight the intricacy of human migration, where migration and mating may not necessarily be random, leaving uncertainty regarding the demographic processes maintaining spatial genetic structure — a concept we revisit below.

When researching the Holocene spatiotemporal dynamics of human genetic diversity and inter-regional mobility across the Eastern Mediterranean and a wider span of Southwest Asia, Koptekin et al. [91] observe since approximately 6000 BP a pattern of decreasing inter-regional *F*_ST_ extending to the present day coupled with increasing inter-regional genetic distance (1 − outgroup *f*_3_). To explain these observations, the authors propose an expanding-mobility model consisting of two sequential historical processes. First is that the observed decrease in inter-regional *F*_ST_ can be attributed to heightened mobility within Southwest Asia and the Eastern Mediterranean in the wake of the Neolithic expansion. However, from approximately 6000–4000 BP onwards, populations in Southwest Asia and the East Mediterranean experienced varying degrees of gene flow from areas beyond their borders, exemplified by the influx of Steppe-related ancestry in the Aegean, South Caucasus, and the Levant, as well as South-Asian-related and West-Siberian-related ancestry on the Iranian plateau.

#### Detecting mobility through modeling shifts in spatio-temporal genetic ancestry

Given adequate sample sizes, the extraction of DNA from skeletons with precise spatial–temporal data promises the possibility of creating a multi-dimensional map illuminating shifts in genetic ancestry over space and time. Genetic differences between populations are expected to correlate with both temporal and spatial distances, with the strength of correlation dependent on mobility levels and spatial population structure. Loog et al. [92] argued that the strength of correlation between genetic differences and spatial or temporal factors will depend on mobility levels: low mobility (strong spatial structure) would lead to stronger space-based correlations, while high mobility would cause time to explain a larger proportion of differences due to its homogenizing effects across space. Leveraging this relationship, Loog et al. [92] developed a scaling factor value (*S*_max_) that can identify relative periods of high mobility by maximizing the correlation between a matrix of genetic differences and a Euclidean matrix of spatial and temporal distances. Using measures of pairwise heterozygosity between approximately 300 genome-wide data covering a time period from the beginning of the Upper Paleolithic to the Iron Age, Loog et al. [92] found at least three distinct stages of high mobility. Whilst identifying relative high mobility as a result of the Neolithic expansion, consistent with previously described ancient DNA data and archaeological findings, their analysis revealed that mobility among Holocene farmers in Europe significantly exceeded that of European hunter-gatherers both before and after the Last Glacial Maximum, suggesting that hunter-gatherers moved either less frequently or over shorter distances compared to farmers.

Leveraging the recent rapid increase in genome-wide ancient DNA, Schmid and Schiffels [93] created an interpolated spatiotemporal ancestry field from which they can then study individual-level mobility through time. Applying their method to 3,138 ancient DNA samples from Western Eurasia, they first use multidimensional scaling (MDS) to capture the two axes that explain the greatest genetic diversity within their sample set and model these as dependent variables in a Gaussian Process Regression analysis with input variables describing the position of each sample in space and time. As the genetic axes stem from MDS analysis, the “ancestry” field in this context does not refer to an admixture coefficient, but rather orthogonal ancestry components captured in the MDS analysis. From this, they derive individual-wise mobility on a large scale by generating a probabilistic similarity search algorithm to determine the likelihood to observe a sample’s MDS coordinates at a certain point in space and time, and the relative spatial distribution of similarity probabilities in a given timeframe.

In addition to capturing previously known major Holocene migration events, such as the Neolithic demographic expansion and the arrival of Steppe ancestry in the third millennium, by considering the spatiotemporal nature of genetic ancestry, Schmid & Schiffels [93] approach highlights the fluidity of ancestry, dispelling notions of the geographically imputable nature of one’s genetic legacy. For example, tracing the spatiotemporal ancestry of Stuttgart, an early Neolithic sample from Central Europe, reveals a progression of highest similarity from the Levant (~ 7500 BC) to Anatolia (post–7000 BC), and then to western Anatolia (6750 BC), reflecting early population structure and migration in the Near East following the Last Glacial Maximum [89]. By 5250 BC, approximately the time of Stuttgart’s death, the peak similarity area includes its burial location in Central Europe, confirming the arrival of Near Eastern ancestry in the region.

Beyond genetic and spatial–temporal information, the incorporation of climatic and environmental data may yield additional insights when interpreting patterns of past mobility. To this end, Racimo et al. [94] developed a spatiotemporally explicit hierarchical Bayesian model to better understand the relationships between changes in climate, ancestry, and paleovegetation. Their model incorporated an interpolated map of major European ancestral components (Mesolithic hunter-gatherers, Neolithic farmers, and Yamnaya Steppe peoples) derived from Ohana [95], simulation-based Holocene paleoclimate reconstructions from PaleoClim [96], and paleovegetation records documenting various land cover types. Notably, they found that the Neolithic farmer migration, characterized by a two-pronged wavefront, did not strongly correlate with vegetational landscape alterations, whereas the faster Early Bronze Age Steppe migration coincided with substantial changes in vegetation, such as the reduction of broad-leaf forests and expansion of pasture lands throughout Europe.

### The question of sample representativeness

Analyses of ancient DNA often rely on small sample sizes, especially in harsh environments that rapidly deteriorate DNA, or when attempting to reconstruct fine-scale spatio-temporal admixture histories where the density of archaeological samples is low. While Reich et al. [40] developed an unbiased estimator to address the challenges of computing the *f*_2_-statistic from small samples, the question remains as to how accurately a single, or small number of samples, can recover the admixture history of an entire ancient population or accurately represent true ancestral admixing sources. This is especially pertinent given that the true admixing sources are highly likely to originate from entirely different ancient communities than those excavated and sequenced. Importantly, the relevance of sample representation is context-dependent, varying with the specific research questions. For instance, Li and Durbin [97] demonstrated that even a single human diploid genome can offer valuable insights into the timing of population divergence, underscoring the breadth of genetic history contained within a single genome due to the processes of recombination and inheritance. In the context of ancient DNA analysis, benchmarks from simulated pulse-admixture and random mating demographic models demonstrate that while limited sample sizes diminish qpAdm’s power, they do not generate bias in the estimation of population admixture [53]. However, under the specific condition of genetic asymmetry between the tested and true sources in two-source qpAdm models, there does appear to be a slight upward bias towards the test population most closely related to the true admixing population [47].

Questions regarding sample representativeness also extend to how comprehensively geographical regions are sampled and subsequently incorporated as candidate sources when investigating a target population’s ancestry through qpAdm analyses. In addressing this issue, through progressively testing more complex qpAdm models until finding a non-rejected model with feasible admixture proportions — a common approach in literature Flegontova et al. [76] found that when the landscape in the vicinity of the target population is poorly sampled, analyses conclude with more complex models (four-way versus two-way). This outcome leads to misleading interpretations because only symmetrically arranged demes yield non-rejected two-way models, while complex models often face rejection due to uncertainty in estimating admixture fractions from similar sources.

The representativeness of ancient DNA samples is further challenged by the extent to which inferences about migration from a limited number of individuals can be reliably extrapolated to broader archaeological contexts, geographical regions or time-periods. Indeed, this sentiment is captured by Sikora et al. [83], commenting on the early discovery of a genetic affinity between present-day Sardinians and a 5300 year old mummy (the Tyrolean Iceman) located near the Austrian-Italian border [98], stating that “… this finding was hindered by the question of how representative this single individual really was for the Central Alpine region”. These challenges are not limited to inferences that cross millenia. In researching the purported “Sea-Peoples” migration hypothesis using ancient DNA extracted from the ancient Levantine archaeological site, Ashkelon, Feldman et al. [99] discovered a shift in the genetic ancestry between the Iron Age I (ASH_IA1) and II (ASH_IA2) periods whereby *f*_4_-statistics of form *f*_4_(ASH_IA2, ASH_IA1; Test, Mbuti) are all significantly negative (*Z*-score ≤ − 3) when Test populations are from either Europe or Anatolia. In explaining the transient nature of the European-related genetic affinity, Feldman et al. [99] state that it could have been diluted by admixture — either the local Ashkelon population or by a gene flow from a closely related population outside of Ashkelon as *f*_4_-statistics of form *f*_4_(ASH_IA2, ASH_LBA; Test, Mbuti) (|*Z*-score|< 2.8) confirmed the genetic symmetry between the Iron Age II and Late Bronze Age Ashkelon cohorts. Given the distinct archaeological contexts of the Iron Age I (infant burials under house floors on the central mound) and Iron Age II samples (cemetery adjacent to the city wall), it is also plausible that these represent coexisting — yet temporally unsampled — ancestries in Ashkelon, obviating the need for a dilution/migration explanation to account for differences in European-related ancestry. Acknowledging the crucial role of comprehensive genetic sampling in historical inquiries, the authors caution that insufficient sampling can lead to erroneous conclusions.

**Fig. 4:**
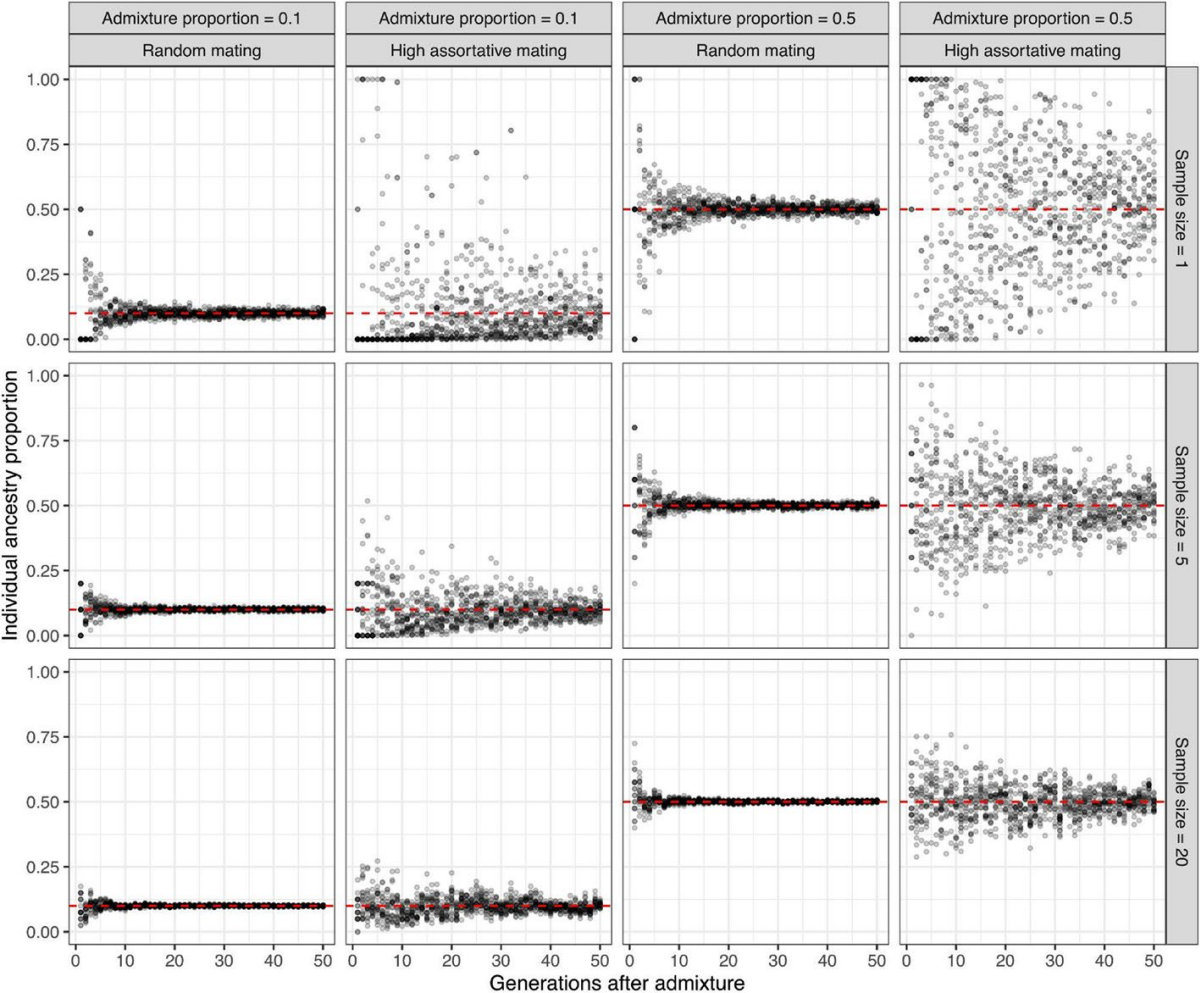
The impact of deviations from random mating and sample sizes on admixture proportions. This SLiM model simulates assortative mating in a non-Wright-Fisher population with a constant size of 2000 individuals, focusing on genomic ancestry over 50 generations. The model represents a Full human genome with a recombination rate of 1 cM/Mb. At the outset, a proportion of individual genomes are tagged with ancestry markers, reflecting immigrant ancestry from a single admixture pulse. Mating is then based on ranked individual ancestry proportions, creating assortative mating according to a specified ancestry correlation between mates [111]. We simulate two scenarios: one with random mating (correlation = 0) and another with strong assortative mating (correlation = 0.9). The simulation tracks changes in individual ancestry proportions across generations

The prospect of contemporaneous groups residing in the same ancient settlement whilst possessing distinct ancestries accentuates the impact of sample partiality on admixture estimation and interpretation in archaeogenetic research. Sociological barriers to human interaction, whether they be economic, class, religious, linguistic, or ethnic, results in deviations from random mating and leads to population structure. Under random mating, admixed genomic segments quickly distribute evenly throughout the population whereby even single samples taken after five or so generations after admixture provide a close estimate of the true population ancestry proportion (Fig. 4). Conversely, non-random mating, particularly positive assortative mating for ancestry-associated traits at the time of admixture [100], maintains a wider spectrum of individual ancestry proportions for longer in a population due to increased pairings between individuals with similar ancestral backgrounds (Fig. 4). The heightened variance in ancestry increases the likelihood of small samples producing estimates that deviate strongly from the population-wide average (Fig. 4). Sampling multiple individuals can alleviate but not completely eliminate this issue (Fig. 4), particularly if archaeological sampling is not completely random and ancestry estimation uncertain due to low data quality. Despite, to our knowledge, there are no systematic studies of assortative mating in ancient populations, historical evidence indicates the presence of segregated communities within ancient societies [101, 102]. In addition, historical records document the presence of external forces shaping societal mobility, such as the Assyrian Empire which is thought to have deported approximately 4.5 million people between 830 and 640 BC, leading to significant linguistic, social, and cultural changes beyond their initial political and economic goals [103, 104]. While identifying any association between ancient societal stratification and ancestry will first require a site-by-site analysis, the impact of burial practices and conditions on DNA preservation [105–107] offers one conceivable mechanism through which societal stratification could distort the perception of population ancestry and migration interpretation — such as socioeconomic status through kinship structures [108–110] potentially being linked to sample survivorship bias. More broadly, skeletal preservation may be differentially impacted by certain funerary practices — such as sub-aerial exposure — resulting in systematic sampling biases distorting the picture of ancient community structure.

## Prospects for the future use of ancient DNA to study human admixture and mobility

Ancient DNA research has already revolutionized our understanding of human mobility, but its full potential is yet to be realized. Future advancements in sampling density (both temporally and geographically), increasing data quality, new analytical techniques, and innovative interdisciplinary collaborations promise to provide a more comprehensive and holistic picture of ancient societal structures and evolution.

### Data advancements

The availability of low-coverage whole-genome sequencing (WGS) imputation methods [112–115], in addition to the growing research into the accuracy and performance of imputation and phasing of low-coverage ancient genomes [116–122], are enabling the prospect of applying powerful demographic inference techniques. These include methods based on the SFS, complete genotype-haplotype information, and recombination-aware, to ancient samples with coverage as low as 0.5 ×, provided an adequate reference panel exists. Indeed, examples of innovative approaches and methods utilizing WGS imputed ancient genomes are beginning to emerge [89, 123–127]. A notable example is the newly developed method twigstats [77], that leverages *f*_2_-statistics estimated from temporal subsets of inferred genome-wide genealogies [128] to enhance qpAdm’s power and has demonstrated promise for uncovering fine-scale population structure in ancient populations.

Recent methodological advancements (ancIBD Ringbauer et al. [126] and IBDSEQ (Browning and Browning [129]) have taken advantage of genome imputation and phasing, making it possible to identify long stretches of the genome shared by pairs of ancient individuals that result from recent genealogical ancestry — so-called identity-by-descent (IBD) segments. Because IBD segments are shared stretches of DNA preserved without intervening recombination events they exhibit an inverse relationship between their length and generational distance from common ancestors, with longer segments signifying recent shared ancestry while shorter fragments reflect more ancient genealogical connections. IBD assessments provide an important complementary approach to allele frequency-based methods in migration studies by detecting direct shared genealogical ancestry between individuals from different archaeological sites. As such, they capture direct signatures of migration-induced relationships between people from distinct homelands, rather than modeling migration using ancient populations as putative proxy sources only indirectly related to the true admixing populations by way of allele frequency similarities [130].

Moreover, with advancements in methods developed to harness inferred genome-wide genealogies for mapping the geographical distribution of genomic ancestors [131, 132], the prospective incorporation of ancient DNA into these approaches promises to dramatically increase our spatiotemporal resolution of genetic ancestry. However, given the prevalence of hybridization capture in ancient DNA generation, evaluating the potential downstream biases of imputation on capture data [116, 117, 126] from diverse geographical origins and underrepresented ancestries in reference panels will be crucial, both for comparative analyses with existing datasets and for samples where economic or technical constraints preclude shotgun sequencing at coverage levels adequate for accurate imputation.

### Interdisciplinary developments

The disparity between archaeological approaches to researching migration, which emphasize the movement of individuals, and ancient DNA, which utilize genetic measures expected from idealized theoretical populations, underscores a fundamental challenge in synthesizing their inferences for describing past demographic events. Moreover, diverse migration scenarios, including single large pulse events, continuous small-scale migration, population size fluctuations, subsequent dilution by different migrant groups, and differential reproductive success due to cultural or genetic advantages, can all potentially affect the ancestry proportions within a population, underscoring the complexity of inferring specific historical demographic processes from ancient DNA data alone. Despite the capacity to detect major genetic shifts indicative of “mass-migrations,” ancient DNA methods cannot translate these findings into specific census counts of individuals who migrated and integrated into new societal contexts. As such, the use of admixture proportions to infer the underlying generative demographic processes presents a fundamental challenge in archaeogenetic research, prompting inquiries into the utility of these estimations for informing our understanding of the societal mechanisms underlying demographic history.

The value of integrating multiple lines of evidence to elucidate human mobility patterns has been demonstrated by a number of paleo/archaeogenomic analyses [133–136]. In particular, the co-analysis of ancient DNA with coarse geographically informative markers, such as stable isotopes, has proven insightful for interrogating signatures of migration covering multiple temporal scales — such as revealing the complex interplay between decreasing generational mobility and the formation of a mixed Anatolian, Levantine, and Iranian/Caucasus ancestry profile in the Anatolian Pre-Pottery Neolithic samples from Nevalı Çori [133], and the inference of state-sponsored resettlement in the Chincha Valley during the pre-Colonial Andes revealed through the co-analysis of textual sources, textile analysis, strontium isotope data, ceramic evidence, and ancient DNA [135]. In addition, the co-analyses of IBD networks and archaeological status markers (such as belt sets for males and coat clasps for females) revealed that female mobility between communities was the main driver of genetic connectivity, with distinct marriage networks maintaining genetic barriers between sites, while individuals with high-status grave goods showed 1.27–2.55 times higher probability of having genetic connections to others, demonstrating a direct correlation between social status and biological relatedness [130].

Spatio-temporal simulations have emerged as a crucial tool in developing population genetic inference [137–143]. In particular, agent-based spatio-temporal modeling incorporating interdisciplinary data has emerged as a powerful approach for unraveling the complex demographic dynamics underlying migration patterns. A compelling example of this methodology is the study by LaPolice et al. [144], who modeled the Neolithic expansion to investigate the interplay between demic and cultural mechanisms in the spread of cultural practices. Their study revealed a counterintuitive relationship between cultural transmission rates and genetic ancestry patterns, demonstrating that a wide range of low but sufficient learning rates can result in predominantly demic diffusion of culture without causing a turnover in genetic ancestry. This finding challenges simplistic interpretations of genetic data alone and highlights the importance of considering multiple factors when studying ancient population movements. Such modeling approaches offer a promising avenue for deepening our understanding of the sociocultural dynamics that shape human migration and genetic diversity.

The emergence of paleo/archaeogenomics is catalyzing a paradigm shift in our understanding of human migration. This interdisciplinary field, which integrates insights from population genetics, archaeology, ancient history, and other disciplines, is not only enhancing our knowledge of past population movements but also paving the way for new theoretical frameworks — such as new conceptual models of intergenerational material cultural transmission [145], and furthering our conceptions of the relationship between material cultural nomenclature and genetic clustering of ancient communities [38]. Looking forward, the synthesis of advancing data-generation techniques, analytical methods, and transdisciplinary integration holds the key to unlocking the true promise of paleo/archaeogenomics in painting the nuanced picture of societal dynamics throughout human history.

## Supplementary Information

Additional file 1: Supplementary Note 1. Introduction to population genetic concepts Supplementary Note 2. Mathematical derivation of the f4-statistic as composed of as combinations of f2-statistics Supplementary Note 3: Implementation of qpAdm in studying ancient human genetic admixture Fig S1. Expected f-statistics under one dimensional stepping stone and hierarchical stepping stone models.

## Data Availability

All scripts used for simulation generation and plot creation are publicly accessible through our GitHub and Zenodo repositories [146, 147] and distributed under the Creative Commons Attribution 4.0 International licence.
