# Publisher Correction: The genomic footprints of migration: how ancient DNA reveals our history of mobility

**DOI:** 10.1186/s13059-025-03706-3

**Published:** 2025-10-20

**Authors:** Matthew P. Williams, Christian D. Huber

**Affiliations:** Department of Biology, The Pennsylvania State University, University Park, PA 16802 USA

**Genome Biology 26, 206 (2025)**

**https://doi.org/10.1186/s13059-025-03664-w**

Following publication of the original article [1], the authors identified numerous misnumbered citations in the text. These numbering issues, are as a result of a typesetting error. The authors have also flagged a minor issue in the supplementary file, page 11, where the sentence currently reads: “Flegontova et al. [9] provide several strategic population selection recommendations: (1) ensure right-group populations temporally post-date or coincide with left-group populations.” This should be corrected to: “Flegontova et al. [9] provide several strategic population selection recommendations: (1) ensure right-group populations temporally pre-date or coincide with left-group populations.” The original article [1] has been corrected.

## Supplementary Information

Additional file 1: Supplementary Note 1. Introduction to population genetic concepts Supplementary Note 2. Mathematical derivation of the f4-statistic as composed of as combinations of f2-statistics Supplementary Note 3: Implementation of qpAdm in studying ancient human genetic admixture Fig S1. Expected f-statistics under one dimensional stepping stone and hierarchical stepping stone models.
