# Supplementary material for: The genomic footprints of migration: how ancient DNA reveals our history of mobility
Source: Genome Biol. 2025 Jul 16;26:206. doi: 10.1186/s13059-025-03664-w (PMC12265385; doi:10.1186/s13059-025-03664-w)
Supplement: Supplementary file 1 — Additional file 1: Supplementary Note 1. Introduction to population genetic concepts Supplementary Note 2. Mathematical derivation of the f4-statistic as composed of as combinations of f2-statistics Supplementary Note 3: Implementation of qpAdm in studying ancient human genetic admixture Fig S1. Expected f-statistics under one dimensional stepping stone and hierarchical stepping stone models. [file 13059_2025_3664_MOESM1_ESM.pdf]

The genomic footprints of migration: how ancient DNA reveals our history of mobility

Supplementary Notes and figures

Matthew P. Williams and Christian D. Huber

Contents

Supplementary Note 1: Introduction to population genetic concepts....2
Allele frequency....2
Coalescence....2
Genetic drift....2
Incomplete lineage sorting....3
Loci....3
Phylogeny, branches, and branch lengths....3
Population structure....3
Supplementary Note 2: Mathematical derivation of the f4 statistic as composed of as combinations of f2-statistics....4
Supplementary Note 3: Implementation of qpAdm in studying ancient human genetic admixture....5
Selection of putative source (left-set) populations....9
Selection of putative reference (right-set) populations....10
Fig S1: Expected f-statistics under one dimensional stepping stone and hierarchical stepping stone models....12
References....13

Supplementary Note 1: Introduction to population genetic concepts

Population genetics provides the fundamental mathematical and conceptual framework through which evolutionary processes can be rigorously studied–encapsulated in Michael Lynch's Indiana University Bloomington research group motto – "Nothing in evolution makes sense except in the light of population genetics" [1] – a witty take on Theodosius Dobzhansky's 1973 famous quotation. To enhance accessibility of our manuscript for readers with limited familiarity with population genetics, we incorporate concise explanations of relevant concepts used throughout the manuscript. Our aim is not to provide a comprehensive treatise of our selected population genetic concepts, but rather that we can provide a sufficient background for all readers such that all can follow our discussion of admixture signatures in ancient DNA and meaningfully be part of the conversations about their application in studying human migration history.

Allele frequency

Allele frequency is the proportion at which a specific genetic variant (allele) occurs within a population. Alleles are different versions of the same gene or DNA sequence arising from mutations. In ancient DNA and population genetics research, these are commonly studied as single nucleotide polymorphisms (SNPs), where a single DNA base pair differs between individuals.

Coalescence

Coalescence is a concept in population genetics where genetic lineages, when traced backwards in time, eventually merge at a common ancestral DNA sequence. When examining a particular position in the genome across different individuals or populations, these sequences originated from a single ancestral sequence at some point in the past from which they all descend. The time at which these lineages merge, or 'coalesce,' can reveal important information about population history and genetic relationships.

Genetic drift

Genetic drift refers to the random fluctuations in allele frequencies that occur in populations over time, simply due to chance events. Since populations have limited sizes, not all individuals contribute equally to the next generation, randomly causing some genetic variants to become more common or rare. Importantly, the effects of genetic drift are most pronounced in small populations.

Incomplete lineage sorting

Incomplete lineage sorting occurs when the evolutionary history of a specific genetic region differs from the overall population history. This happens because different genes can have different evolutionary paths due to random inheritance patterns. For example, some present-day humans may share genetic history at specific loci more recently with Neanderthals than other present-day humans simply due to chance, despite present-day humans overall being more closely related to each other than we are to Neanderthals.

Loci

Loci (singular: locus) are specific positions or locations on chromosomes. In population genetics, these often refer to the positions where genetic variations such as SNPs occur. These locations are the genetic data on which we study genetic variation across populations.

Phylogeny, branches, and branch lengths

A phylogeny (also called a phylogenetic tree) is a branching diagram that depicts how populations are related through shared ancestry. The connecting lines in this tree are called branches, with each branch representing an evolutionary lineage. Terminal branches, or leaves, represent sampled or present-day populations. The length of each branch corresponds to the amount of genetic change (often represented as units of genetic drift measured with the f_2-statistic) that occurred along that lineage.

Population structure

Population structure refers to the pattern of genetic variation that arises when a population deviates from random mating, resulting in smaller subgroups with limited genetic exchange between them. Instead of random mating across the entire population, individuals tend to mate within their subgroups, leading to genetic differentiation between these subgroups. As a result, individuals within the same subgroup share more recent common ancestors (more recent coalescence events) across their genome compared to individuals from different subgroups.

Supplementary Note 2: Mathematical derivation of the f_4 statistic as composed of as combinations of f_2-statistics

The f_2-statistic quantifies genetic drift between two sampled populations by measuring the average squared difference in their allele frequencies. Alternatively, the f_4-statistic measures the covariance in allele frequency differences between pairs of populations. Below, we algebraically show how the f_4-statistic is composed of combinations of f_2-statistics using populations P1–P4, with their corresponding allele frequencies (p_1, …, p_4). The f_4-statistic f_4(P1, P2; P3, P4) measures the covariance in allele frequency differences between (P1-P2) and (P3-P4) with expectation (average) over many SNPs. If populations (P1 and P2) and (P3 and P4) are indeed clades, then the expectation of f_4(P1, P2; P3, P4) is zero because the allele frequency differences are independent of each other and therefore the covariance is zero. However, in the presence of admixture between two populations, say in the specific case of admixture from P2 to P3, the amount of genetic drift separating them is reduced, such that f_2(P1, P3) > f_2(P2, P3), resulting in a negative f_4-statistic from the vantage of the f_2-statistics. From the vantage of the f_4-statistic, admixture between P2 and P3 introduces covariance in the difference in genetic drift between (P1 and P2) and (P3 and P4) as their allele frequencies are no longer independent of each other.

$$f_4(P_1, P_2, P_3, P_4) = \mathbb{E}[(p_1 - p_2)(p_3 - p_4)]$$

$$= \mathbb{E}[p_1p_3 - p_1p_4 - p_2p_3 + p_2p_4]$$

$$= \frac{1}{2}\mathbb{E}\left[-p_1^2 + 2p_1p_3 - p_3^2 + p_1^2 - 2p_1p_4 + p_4^2 - p_2^2 - 2p_2p_3 + p_3^2 - p_2^2 + 2p_2p_4 - p_4^2\right]$$

$$= \frac{1}{2}\left(-\mathbb{E}[(p_1 - p_3)^2] + \mathbb{E}[(p_1 - p_4)^2] + \mathbb{E}[(p_2 - p_3)^2] - \mathbb{E}[(p_2 - p_4)^2]\right)$$

$$= \frac{1}{2}\left(F_2(P_1, P_4) + F_2(P_2, P_3) - F_2(P_1, P_3) - F_2(P_2, P_4)\right)$$

Supplementary Note 3: Implementation of qpAdm in studying ancient human genetic admixture

qpAdm has become a mainstay in ancient-DNA research with early conceptual iterations first introduced in Reich et al. [2], Moorjani et al. [3], and Lazaridis et al. [4]). Here we present an introductory guide to its framework and methodology – how qpAdm builds and tests admixture models to estimate ancestry proportions and compute a statistical test for how well that model fits the genetic data. For a technical description of the qpWave/qpAdm algorithmic and computational details, we recommend to readers the Supplementary Information section 12 of Lazaridis et al. [4], Supplementary Materials sections 9 and 10 of Haak et al. [5], Supplementary Materials 2 of Harney et al. [6], a Documentation for qpWave and qpAdm written by Nick Patterson housed in the Institut Pasteur gensoft repository (https://gensoft.pasteur.fr/docs/AdmixTools/7.0.2/pdoc.pdf), and the online documentation of qpAdm implementations in the R software packages admixR (https://bodkan.net/admixr/articles/vignette-02-qpAdm.html) [7] and admixtools2 (https://uqrmaie1.github.io/admixtools/articles/qpadm.html) [8]. Throughout this guide we will periodically illustrate the qpAdm concepts using "Model 1" from Fig. 1A of the main manuscript. A brief clarification regarding qpAdm terminology at the outset is warranted. In Haak et al.'s [5] initial formal implementation of qpAdm, the source populations for whom one was testing were designated "references" while Right-set populations were termed "outgroups." Subsequently, Harney et al. [6] revised this nomenclature, redesignating "references" as source populations and renaming "outgroups" as the right-group set (the convention adopted here), recognizing that these populations do not serve as true phylogenetic outgroups, which would necessitate equal genetic distances to all source and target populations – a condition violating fundamental qpAdm assumptions (further outlined below).

The fundamental data structure underlying qpAdm to both test admixture models and estimate ancestry contributions from candidate source populations is a matrix **X** formed by empirical f_4-statistics (that is, a matrix estimated from f_4-statistics from sample allele frequencies as opposed to the theoretical f_4-statistic matrix **X**). In a typical scenario, a user supplies the following:

- A target population whose history is of interest, T
- A candidate list of putative source populations, $S_1,S_2,\ldots,S_{a-1}$
- A set of reference populations, $R_0,R_1,\ldots,R_{b-1}$

In qpAdm these populations are then used to form two separate groups, a left and right-set. The left-set comprises the target and source populations with a combined total of *a* populations in the set and the right-set comprises the reference populations with a total of *b* populations in the set. From these population sets, qpAdm fixes L_0, i.e., the target (T) and

one population from the right-group R_0 – in practice typically the population with the highest genetic coverage – in every statistic of form $f_4(L_0, L_i; R_0, R_j)$ for $i \in (1, 2, \ldots, a - 1)$ and $j \in (1, 2, \ldots, b - 1)$ yielding a matrix $\widehat{X}$ of dimensions $(a - 1)\times(b - 1)$. For concreteness, using the Model 1 demography, T = PX, sources = (P1, P2) and references = (P3, P4, P5, P6, PO), with the left-set = (T, P1, P2) and the right-set = (P3, P4, P5, P6, PO), the resulting f_4-statistic matrix $\widehat{X}$ will have 2×4 = 8 f_4-statistic elements (two rows for sources P1 and P2, and four columns, P3-P6 when PO is fixed as R_0).

There are summaries and calculations on matrices developed from linear algebra that can be informative for making inferences about population relationships beyond interrogating individual f_4-statistics. One such summary is the rank of a matrix (r) which is the maximum number of linearly independent rows or columns in a matrix. In practice, the number of right-group populations should be larger than the number of left-group populations ($b > a$) which entails that the maximum possible rank of the f_4-statistic matrix is $(a - 1)$. Importantly, it was first demonstrated in Reich et al. [2] that the minimum number of gene-flow events needed to explain shared drift between left and right-group sets of the theoretical f_4-statistic matrix **X** (i.e., comprised of the expected f_4 values without measurement error) is $r + 1$ (more concretely, if **X** has rank r, and there are n independent gene flow events linking the right-and-left-group sets then $r+1 \leq n$). As such, because the rank of an f_4-statistic matrix is informative about the number of independent gene-flow events connecting the left and right-group sets, testing its rank under various population set configurations can be leveraged to inform about the genetic history separating sets of populations.

In practice, f_4 values are estimated with measurement error, which leads to the empirical $\widehat{X}$ matrix having full rank of $(a\text{-}1)$ due to the random deviations making all columns linearly independent. To test if the underlying expected f_4-statistic matrix has rank (r)–i.e., the "simpler" model constitutes the null hypothesis whereas the alternate model represents at least r+1 gene flow events–qpAdm forms a simplified r-rank version of the $\widehat{X}$ matrix and uses a statistical test to determine if it explains the data as well as a matrix of rank r+1. The form of the simplified r-dimensional matrix is $A.B$; where A is $(a - 1)\times r$ and B is $r\times(b - 1)$. For clarity, taking one rank less than the full rank (i.e., a - 1), we have $A = (a - 1)\times((a - 1) -1)$ and $B = ((a - 1) - 1)\times(b - 1)$.

How well the $A.B$ matrix approximates values in the original f_4-statistic matrix $\widehat{X}$ is captured in the residual matrix, $E = \widehat{X} - AB$ where the residual for cell (i, j) is $D(i, j) = \widehat{X}(L_i, R_j) - (A \cdot B)_{i,j}$, giving the distance between the observed and predicted f_4-statistic values. A matrix of full rank will have $E = 0$, i.e. it will perfectly reconstruct the original f_4-statistic

matrix; therefore, if a lower-rank A·B approximation still yields only small residuals; it indicates that the matrix rows (and thus sources) in $\widehat{X}$ are not fully independent but instead carry redundant genetic information–with the assumption that this reflects their shared demographic or evolutionary history linking them to the right-group populations. However, if there are large residuals, then this suggests that the f_4-statistic matrix, $\widehat{X}$ can't be well approximated by a lower rank r matrix AB, and that as few as r independent gene-flow events does not adequately explain the genetic relationship between the left and right-group sets. This indicates that the matrix rows (sources) are largely independent in their drift relationships to the right-group populations, implying that the true rank of $\widehat{X}$ is larger than r and not reducible to r dimensions.

We note that the f_4-statistic matrix, $\widehat{X}$, is first computed on a genome-wide average. However, the demographic history for sites along the genome are not completely independent of each other and there is expected to be correlation in the genealogical history for SNPs that are physically close to each other–so called linkage disequilibrium (LD). As such f_4-statistics computed at nearby positions in the genome will possess covariance for their estimated value. To account for this, qpAdm employs a block jackknife approach whereby the genome is split into blocks of a length that is long enough to both take into account linkage between sites and leave enough blocks to perform an adequate number of tests with. This jackknife then sequentially computes the f_4-statistic on all remaining blocks in the genome, leaving out one block at a time. In empirical ancient human DNA research, the block size is commonly taken to be 5 centimorgans (cM) – a statistical genetics measure for the probability two alleles on a chromosome are inherited together–which, on the 1240K dataset, results in approximately 700 blocks. The role of the block jackknife is to provide some measure about the uncertainty of each f_4-statistic entry in $\widehat{X}$. In addition to that, it also provides information about the covariance of errors between different f_4-statistics. qpAdm uses the block jackknife approach to generate an estimate of the error covariance of the f_4-statistic matrix $\widehat{X}$ and then it takes the inverse of the error covariance matrix to form $Q = W^{-1}$, which is a measure of the precision of the f_4-statistic estimates.

qpAdm uses these error covariances $Q(i, j)$ to weight the $\widehat{X}$ - AB residual for cell $D(i, j)$, to compute the likelihood of the model with the assumption that the errors are multivariate normally distributed–which is calculated as the weighted sum of squared residuals and evaluated via a likelihood-ratio test between models of different ranks (each rank corresponding to a hypothesized number of independent gene-flow events with the lower rank functioning as the null hypothesis). The likelihood-ratio test yields a χ^2 distributed statistic ($\mathcal{L}$) whose degrees of freedom depend on the rank (r) difference

between the two tested models. A non-significant p-value (commonly taken at 0.01 and 0.05) implies rank *r* suffices; whereas a significant p-value implies additional independent gene flow events are required to explain the relationship between the two sets of populations. Importantly, extensive simulations in Harney et al. [6] have shown the LRT to perform well in identifying the true rank of the f_4-statistic matrix ($\widehat{X}$).

It is important to note here that the rank of $\widehat{X}$ does not carry any inherent information about the specific gene-flow events introducing independent genetic drift, nor the populations involved. However, if the rank (*r*) of the matrix when including the target population with the source populations is statistically significantly greater than the rank (*r*) with just the source populations alone, this provides information that there are at least one independent gene-flow events connecting the target population with the right-group set that is not captured by the sources. Under such a scenario, one would reject the model of the source populations (with a typically p-value threshold used in the literature of ≥ 0.05 or 0.01) as capturing the full extent of ancestry of the target population (note that in practise, the qpAdm software will estimate the ancestry contributions regardless of the model statistical test). Recalling that the f_4-statistic matrix $\widehat{X}$ described above is factorized into two smaller matrices, *A* and *B*, where *A* is formed with (a - 1) rows and *r* (rank) columns and captures the genetic relationships between the target and source populations, including the degree to which they possess distinct or shared genetic drift, relative to the right-group populations.

Under an admixture model, if the target population T is truly a linear mixture of the source populations ($L_1, L_2, \ldots, L_{a-1}$), such that the allele frequency in the target (p_T) is sum of the allele frequency of its sources (p_{L1}, $p_{L2}, \ldots p_{La-2}$), weighted by the admixture coefficient (W_{L1}, $W_{L2}, \ldots, W_{La-1}$) of each source such that $p_T = W_{L1}p_{L1} + W_{L1}p_{L2} + \ldots + W_{La-1}p_{La-1}$, then it follows that for a given pair of right-group populations R_1, R_2 the f_4-statistic of the source populations weighted by their ancestry contribution (*w*) to the target equals the f_4-statistic of the target population as a clade; i.e.,

$$\sum_i w_i f_4(T,\ L_i;\ R1,\ R2) \ =\ f_4(T,\ T;\ R1,\ R2) \ =\ 0.$$

This in effect is asking what admixture weights are required to be contributed from the source to the target to equal the f_4-statistic involving the target. In qpAdm the admixture weight-coefficients are estimated by finding the linear combination of the source populations' f_4-statistic patterns that best reconstructs the target population's f_4-statistic pattern. To compute the admixture weight coefficients (w_1, w_2, . . ., w_{a-1}), qpAdm solves the above equation by least squares to minimize the difference between the observed f_4-statistics for the target and those predicted by the model.

We finally note that the block jackknife is also used to compute a covariance matrix of the admixture weight estimates. The standard errors for these admixture weights are derived from this covariance matrix (typically as the square roots of its diagonal elements). These standard errors reflect the variability observed in the f_4-statistics when computed using different genomic blocks.

We now turn to the question concerning the appropriate selection of source and right population sets in qpAdm analyses. As above, we direct readers to the documentation in SI section 9 of Haak et al. [5] and SI section Usage Recommendations of Harney et al. [6] for exemplary guidance. An important aspect of left and right-group population selection is the importance of selecting populations and samples that do not violate qpAdm assumptions–particularly with respect to the rank test assumptions – namely that the outcome of the maximum rank is r+1 is under the assumption of no migration between groups present in the left and right sets. Whilst the fundamental assumptions of qpAdm have been recognized since its introduction, Flegontova et al. [9] have recently rigorously tested it through comprehensive simulations of both admixture graph-shaped and stepping stone migration scenarios. Their results demonstrate that complex migration networks can result in violations of a core qpAdm assumption – that "*there should be no genetic drift shared exclusively by proxy sources and right populations (but not by the target) or shared exclusively by the target and right populations (but not by proxy sources).*" Below, we present a concise summary of the literature's combined principal recommendations.

Selection of putative source (left-set) populations

qpAdm source populations comprise groups putatively ancestral to the target population of interest. Their identification typically emerges from exploratory analyses such as principal component analysis (PCA) or ADMIXTURE that provide some prior evidence suggesting their genetic contributions to the target population, or from archaeological/historical research hypotheses. One of the first principles regarding the selection of which source populations to use among closely related candidate sources, researchers should prioritize populations with larger sample sizes and higher genomic coverage. Importantly, to enhance statistical power, it is possible for individuals from similar archaeological contexts with comparable genetic profiles to be combined into metapopulations. To validate the merging of individuals into metapopulations, researchers can perform pairwise f_4-statistic cladality tests of form $f_4(\mathrm{IND}_1, \mathrm{IND}_2; \mathit{Test}, \mathrm{Outgroup})$, clustering individuals ($\mathrm{IND}_{1,2}$) that fail to reject clade relationships relative to diverse *Test* populations.

For the selection of source populations themselves, researchers can perform pre-screening admixture f_3-statistic tests of form $f_3(\mathrm{Target}; \mathit{Test}, \mathit{Test})$, across a list of putative *Test* sources and retaining only those that produce statistically

significant results. However, the f_3-statistic approach is quite conservative, potentially generating false negatives due to demographic conditions and sampling limitations described in the main text. An additional consideration when selecting source populations is the critical assumption of qpAdm that source populations must exhibit a caudal relationship to the true admixing sources – meaning no gene-flow has entered the source population following its divergence from the actual (true) admixing population. Violations of this assumption through such gene-flow events can elevate the rank of matrix $\widehat{X}$ –particularly if the admixing population's ancestry is present in the right-group set–resulting in statistical rejection of the proposed qpAdm model.

ancestors

Selection of putative reference (right-set) populations

Selecting optimal populations for the right-group set is intrinsically linked to the choice of candidate source populations. Understanding qpAdm's foundational principles – specifically how it identifies independent gene flow events between left and right-group sets using f_4-statistics – helps to illuminate the implications of the method's underlying assumptions.

qpAdm's primary assumption requires that at least one right-group population must exhibit differential relationships to the left populations. This requirement's importance becomes evident when considering that if all right-group populations were equidistant from all left populations (functioning as true outgroups where for instance $f_2(\text{R1, Left}_i) = f_2(\text{R1, Left}_j)$), then f_4-statistics comprising the matrix $\widehat{X}$, such as $f_4(\text{T, L}_i\text{; R0, R1})$ and $f_4(\text{T, L}_j\text{; R0, R1})$, would produce identical statistics. Consequently, matrix $\widehat{X}$ would have rank ($r = 0$) and would lack sufficient resolution in the f_4-statistic matrix to discern evolutionary relationships between source, target, and right-group populations. In the SI section 9 of Haak et al. [5] the authors recommend pre-screening right-group populations using statistics of form $f_4(\text{L}_{1,2,..,a}, \text{R}_k\text{; R}_l, \text{R}_m)$, ideally seeking low correlation between $f_4(\text{L}_1, \text{Right}_k\text{; Right}_l, \text{Right}_m)$ and $f_4(\text{L}_2, \text{Right}_k\text{; Right}_l, \text{Right}_m)$, as high correlation indicates indistinguishable source populations relative to the right-group. Complementarily, SI section Usage Recommendations of Harney et al. [6] advocate pre-screening with f_4-statistics of form $f_4(\text{Left}_i, \text{Left}_j\text{; Right}_k, \text{Right}_l)$, excluding right populations that generate no significant statistics as they lack informative value.

The selection of populations for the right-group set also carries implications for model violations related to the test of the matrix $\widehat{X}$ rank. The qpAdm p-value evaluates whether a model with n independent gene-flow events is preferable to one with at least n+1 events. Gene flow between left and right groups subsequent to the formation of the target population may inflate the inferred number of gene-flow events beyond what accurately explains the target population's ancestry. To

mitigate such violations, Flegontova et al. [9] provide several strategic population selection recommendations: (1) ensure right-group populations temporally pre-date or coincide with left-group populations; (2) ensure that the target group post-dates or coincides with the candidate sources; (3) restrict both left and right-group population sets to subcontinental regions rather than global sampling; (4) prioritize dense sampling within specific geographical regions of interest over sparse sampling across wider areas; and (5) favor analyses where archaeological and historical evidence strongly support the conclusions.

Concerning the number of right-group populations to include, Harney et al. [6] demonstrated through extensive simulation testing that large numbers of right-groups can lead to poorly estimated f_4-statistic covariance matrices, biasing p-values toward zero and increasing false negative rates (rejecting true models). This effect manifests with as few as 35 right-group populations, though the optimal number is contingent on genetic coverage and evolutionary relationships across all populations under analysis. A final word on comparative analyses between different targets and models with respect to the right-group populations. In order that models can be comparable, both between targets and with different candidate sources, recommendation across all qpAdm documentation is that the populations included in the right population set should be as similar as possible.

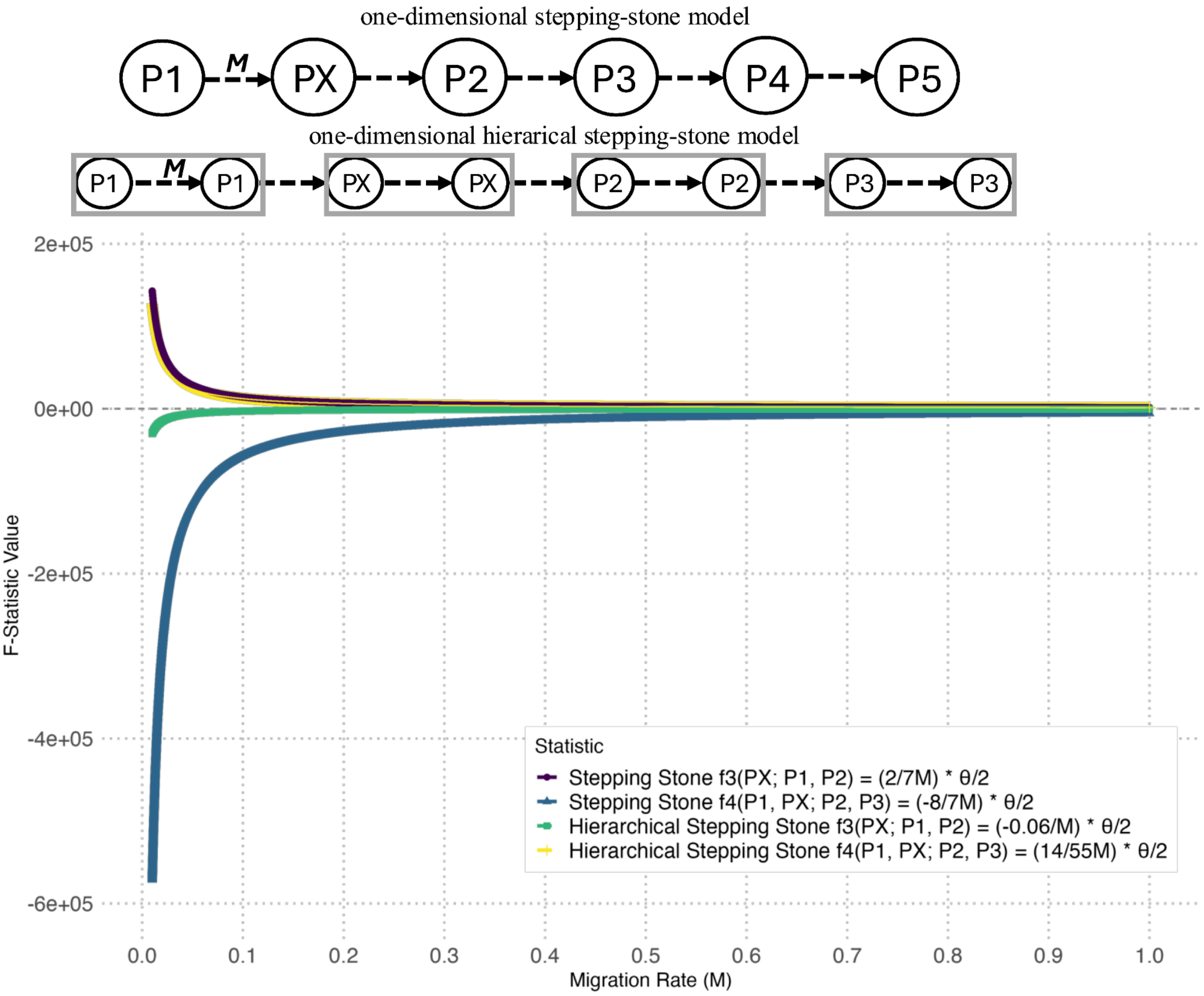

Fig S1: Expected *f*-statistics under one dimensional stepping stone and hierarchical stepping stone models.

F-statistics were calculated following Peter. [10], with θ = 10,000 and 1,000,000 *M* values sampled from the interval [0, 1]. Within the hierarchical model, grey boxes denote the formation of meta-populations from constituent sub-demes.

References

1. Lynch M. The Origins of Genome Architecture. Sunderland, MA: Sinauer associates;2007;389:370.

2. Reich D, Patterson N, Campbell D, Tandon A, Mazieres S, Ray N, et al. Reconstructing native American population history. Nature. 2012;488:370–4.

3. Moorjani P, Thangaraj K, Patterson N, Lipson M, Loh P-R, Govindaraj P, et al. Genetic evidence for recent population mixture in India. The American Journal of Human Genetics. 2013;93:422–38.

4. Lazaridis I, Patterson N, Mittnik A, Renaud G, Mallick S, Kirsanow K, et al. Ancient human genomes suggest three ancestral populations for present-day Europeans. Nature. 2014;513:409–13.

5. Haak W, Lazaridis I, Patterson N, Rohland N, Mallick S, Llamas B, et al. Massive migration from the steppe was a source for Indo-European languages in Europe. Nature. 2015;522:207–11.

6. Harney É, Patterson N, Reich D, Wakeley J. Assessing the Performance of qpAdm: A Statistical Tool for Studying Population Admixture. Genetics. 2021;217:iyaa045-.

7. Petr M, Vernot B, Kelso J. admixr—R package for reproducible analyses using ADMIXTOOLS. Bioinformatics. 2019;35:3194–5.

8. Maier R, Flegontov P, Flegontova O, Işıldak U, Changmai P, Reich D. On the limits of fitting complex models of population history to f-statistics. Elife. 2023;12:e85492.

9. Flegontova O, Işıldak U, Yüncü E, Williams MP, Huber CD, Kočí J, et al. Performance of qpAdm-based screens for genetic admixture on graph–shaped histories and stepping stone landscapes. Genetics. 2025;230:iyaf047.

10. Peter BM. Admixture, Population Structure, and F -Statistics. Genetics. 2016;202:1485–501.
